# Supplementary material for: Nordic study on human milk fortification in extremely preterm infants: a randomised controlled trial—the N-forte trial
Source: BMJ Open. 2021 Nov 23;11(11):e053400. doi: 10.1136/bmjopen-2021-053400 (PMC8611420; doi:10.1136/bmjopen-2021-053400)
Supplement: Supplementary data [file bmjopen-2021-053400supp001.pdf]

Supplementary table 1 Nutritional content of the study product

Nutrient	Humavant+6®	Humavant CR®	Unit
Calories	143.82	262.22	kcal/100 mL
Fat	9.38	25.59	g/100 mL
Protein	5.92	0.82	g/100 mL
Carbohydrates	8.91	6.92	g/100 mL
Saturated fat	3.93	10.15	g/100 mL
Sugar	6.12	4.53	g/100 mL
Vitamins			
Vit A	46.21	114.65	µg/100 mL
Vit D	0.21	0.09	µg/100 mL
Vit K	<5.10	<4.85	µg/100 mL
Vit C	<1.02	<0.97	mg/100 mL
Vit B1, thiamine	5.92	<4.85	µg/100 mL
Vit B2, riboflavin	19.18	11.14	µg/100 mL
Vit B6, pyridoxine	<5.10	<4.85	µg/100 mL
Niacin	0.08	0.06	mg/100 mL
Folic acid	13.06	<2.43	µg/100 mL
Vit B12, cobalamin	<0.14	<0.14	µg/100 mL
Pantothenic acid	0.17	0.15	mg/100 mL
Biotin	<2.55	<3.17	µg/100 mL
Vit E, α-tocopherol	0.37	1.54	mg/100 mL
Minerals			
Salts	326.10	17.27	mg/100 mL
Sodium	130.69	6.77	mg/100 mL
Chloride	101.79	N/A	mg/100 mL
Potassium	187.17	64.83	mg/100 mL
Calcium	339.86	19.82	mg/100 mL
Phosphorus	189.20	2.83	mg/100 mL
Magnesium	21.48	0.02	mg/100 mL
Iron	0.13	0.33	mg/100 mL
Zinc	2.28	0.03	mg/100 mL
Copper	237.50	<0.03	µg/100 mL
Iodine	23.36	19.89	µg/100 mL
Selenium	9.65	2.07	µg/100 mL
Manganese	5.11	<4.03	µg/100 mL
Chromium	1.57	<0.41	µg/100 mL
Molybdenum	2.55	<1.94	µg/100 mL
Fluoride	<0.02	<0.02	mg/100 mL
