# Supplementary material for: Nordic study on human milk fortification in extremely preterm infants: a randomised controlled trial—the N-forte trial
Source: BMJ Open. 2021 Nov 23;11(11):e053400. doi: 10.1136/bmjopen-2021-053400 (PMC8611420; doi:10.1136/bmjopen-2021-053400)
Supplement: Author's manuscript [file bmjopen-2021-053400.draft_revisions.pdf]

BMJ Open is committed to open peer review. As part of this commitment we make the peer review history of every article we publish publicly available.

When an article is published we post the peer reviewers' comments and the authors' responses online. We also post the versions of the paper that were used during peer review. These are the versions that the peer review comments apply to.

The versions of the paper that follow are the versions that were submitted during the peer review process. They are not the versions of record or the final published versions. They should not be cited or distributed as the published version of this manuscript.

BMJ Open is an open access journal and the full, final, typeset and author-corrected version of record of the manuscript is available on our site with no access controls, subscription charges or pay-per-view fees (http://bmjopen.bmj.com).

If you have any questions on BMJ Open's open peer review process please email info.bmjopen@bmj.com

BMJ Open

Nordic study on human milk fortification in extremely preterm infants: a randomised controlled trial: the N-forte trial

Journal:	*BMJ Open*
Manuscript ID	bmjopen-2021-053400
Article Type:	Protocol
Date Submitted by the Author:	11-May-2021
Complete List of Authors:	Jensen, Georg Bach; Linköping University, Department of Biomedical and Clinical Sciences; Linköping University, Department of Paediatrics<br>Ahlsson, Fredrik ; Uppsala University, Department of Women´s and Children´s Health<br>Domellöf, Magnus; Umea University, Department of Clinical Sciences, Pediatrics<br>Elfvin, Anders; Sahlgrenska Academy, Institute of Clinical Sciences, Department of Paediatrics; Sahlgrenska University Hospital, Queen Silvia Children's Hospital, Department of Paediatrics<br>Naver, Lars; Karolinska Institute, Department of Clinical Science, Intervention, and Technology; Karolinska University Hospital, Department of Neonatal Medicine<br>Abrahamsson, Thomas; Linköping University, Department of Biomedical and Clinical Sciences; Linköping University, Department of Paediatrics
Keywords:	Neonatal intensive & critical care < INTENSIVE & CRITICAL CARE, Nutritional support < GASTROENTEROLOGY, NEONATOLOGY, Developmental neurology & neurodisability < PAEDIATRICS

SCHOLARONE™
Manuscripts

I, the Submitting Author has the right to grant and does grant on behalf of all authors of the Work (as defined in the below author licence), an exclusive licence and/or a non-exclusive licence for contributions from authors who are: i) UK Crown employees; ii) where BMJ has agreed a CC-BY licence shall apply, and/or iii) in accordance with the terms applicable for US Federal Government officers or employees acting as part of their official duties; on a worldwide, perpetual, irrevocable, royalty-free basis to BMJ Publishing Group Ltd ("BMJ") its licensees and where the relevant Journal is co-owned by BMJ to the co-owners of the Journal, to publish the Work in this journal and any other BMJ products and to exploit all rights, as set out in our licence.

The Submitting Author accepts and understands that any supply made under these terms is made by BMJ to the Submitting Author unless you are acting as an employee on behalf of your employer or a postgraduate student of an affiliated institution which is paying any applicable article publishing charge ("APC") for Open Access articles. Where the Submitting Author wishes to make the Work available on an Open Access basis (and intends to pay the relevant APC), the terms of reuse of such Open Access shall be governed by a Creative Commons licence – details of these licences and which Creative Commons licence will apply to this Work are set out in our licence referred to above.

Other than as permitted in any relevant BMJ Author's Self Archiving Policies, I confirm this Work has not been accepted for publication elsewhere, is not being considered for publication elsewhere and does not duplicate material already published. I confirm all authors consent to publication of this Work and authorise the granting of this licence.

For peer review only

Nordic study on human milk fortification in extremely preterm infants: a randomised controlled trial: the N-forte trial

Georg Bach Jensen[1], Fredrik Ahlsson[2], Magnus Domellöf[3], Anders Elfvin[4,5], Lars Navér[6,7], Thomas Abrahamsson[1]

[1] Department of Biomedical and Clinical Sciences and Department of Paediatrics, Linköping University, Linköping, Sweden

[2] Department of Women´s and Children´s Health, Uppsala University, Uppsala, Sweden

[3] Department of Clinical Science, Pediatrics, Umeå University, Umeå, Sweden

[4] Department of Paediatrics, Institute of Clinical Sciences, Sahlgrenska Academy, University of Gothenburg, Gothenburg, Sweden

[5] Region Västra Götaland, Sahlgrenska University Hospital, Queen Silvia Children's Hospital, Department of Paediatrics, Gothenburg, Sweden

[6] Department of Clinical Science, Intervention, and Technology, Karolinska Institutet, Stockholm, Sweden

[7] Department of Neonatal Medicine, Karolinska University Hospital, Stockholm, Sweden

Corresponding author:
Thomas Abrahamsson
Department of Biomedical and Clinical Sciences and Department of Paediatrics, Linköping University,
Crown Princess Victoria Children´s hospital
University Hospital
SE-581 85 Linköping
Sweden
thomas.abrahamsson@liu.se
+46-709566815

For peer review only - http://bmjopen.bmj.com/site/about/guidelines.xhtml

ABSTRACT

Introduction
The mortality rate of extremely low gestational age (ELGA) (born < gestational week 28+0) infants remains high, and severe infections and necrotising enterocolitis (NEC) are common causes of death. Preterm infants receiving human milk have lower incidence of sepsis and NEC than those fed a bovine milk-based preterm formula. Despite this, fully human milk fed ELGA infants most often have a significant intake of cow's milk protein from bovine-based protein fortifier. The aim of this study is to evaluate whether the supplementation of human milk-based, as compared with bovine-based, nutrient fortifier reduces the prevalence of NEC, sepsis and mortality in ELGA infants exclusively fed with human milk.

Methods and analysis
A randomised-controlled multi-centre trial comparing the effect of a human breast milk-based fortifier with a standard bovine protein-based fortifier in 222 to 322 ELGA infants fed human breast milk (mother´s own milk and/or donor milk). The infants will be randomised to either fortifier before reaching 100 mL/kg/day in oral feeds. The intervention, stratified by centre, will continue until the target postmenstrual week 34+0. The primary outcome is a composite of NEC, sepsis or death. Infants are characterised with comprehensive clinical and nutritional data collected prospectively from birth until hospital discharge. Stool, urine, blood and breast milk samples are collected for analyses in order to study underlying mechanisms. A follow-up focusing on neurological development and growth will be performed at 2 and 5.5 years of age. Health economic analyses will be made.

Ethics and dissemination
The study is conducted according to ICH/GCP guidelines and is approved by the regional ethical review board in Linköping Sweden (Dnr 2018/193-31, Dnr 2018/384-32). Results will be presented at scientific meetings and published in peer-reviewed publications.

Trial registration number
The study was registered with ClinicalTrials.gov NCT03797157, January 9th, 2019.

STRENGTHS AND LIMITATIONS OF THIS STUDY
- A randomised-controlled multi-centre trial on human milk-based fortification in ELGA infants powered to detect a reduction in sepsis, NEC and mortality as a composite outcome.
- Several assessments of outcomes will be made blinded, such as the radiological assessment of NEC.
- Both study groups will receive exclusively enteral feeding with breast milk and thereby not differ, besides the different breast milk fortification.
- A significant positive result of this trial would justify implementation of human milk-based fortifiers in the neonatal intensive care units.
- Targeted fortification of breast milk and the difference in nutrient content between the study product and the standard fortification made it impossible to blind the study after randomisation.

For peer review only

For peer review only - http://bmjopen.bmj.com/site/about/guidelines.xhtml

INTRODUCTION

Extremely preterm infants are at risk of severe morbidity and mortality

Although neonatal care has improved markedly during the last decades, mortality and morbidity remain high with a 1-year mortality of 23% in ELGA neonates in affluent countries [1]. It is well known that nutrition is a key factor for survival, clinical course and later outcome of high-risk preterm infants; and of particular relevance to this trial is the evidence for beneficial roles of human milk in neonatal care, underpinning current recommendations for its use [2-4]. Extremely preterm infants (born before 28 weeks of completed gestation) are initially fed both enterally and parenterally while enteral feed tolerance develops. The primary choice is the infant's own mother's milk. Preterm formula or donor breast milk can be used when mothers provide insufficient amounts of breast milk, and in the Nordic countries, donor breast milk is routinely used for preterm infants up to 32-34 weeks postmenstrual age. A growing literature supports the use of human milk for feeding preterm infants, in particular for the smallest and most vulnerable preterm infants.

Reduction of necrotising enterocolitis (NEC)

NEC remains a significant problem in neonates with an incidence between 4 to 15% in very low birth weight (VLBW; <1500g) infants [1, 5, 6]. The pathogenesis is multifactorial, and the disease leads to intestinal inflammation, ischemia and necrosis. The main risk factors besides prematurity, are formula feeding, hypoxic–ischaemic injury, and abnormal bacterial gut colonisation [7, 8]. NEC frequently results in death (around 20-30%) or need for surgical resection of necrotic gut which may lead to short bowel syndrome. Further, NEC is a major adverse factor for neurological impairment [9, 10]. The health economic cost of NEC is large [11, 12]. The most recent Cochrane meta-analysis, including 8 randomized controlled trials with a total of 1605 preterm infants, shows that human milk, as compared to formula, reduces the risk of NEC by a factor 1.9 but it is not clear whether this effect is due to protective factors in human milk or the detrimental effects of cow's milk protein in formula [13].

Reduction in neonatal sepsis

Neonatal late onset sepsis remains a life-threatening and common problem in neonatal care. The incidence is 35-40% in Swedish ELGA infants (www.snq.se). Growing evidence links sepsis to the mode of feeding. Even though no significant effect of human milk on sepsis was shown in the latest meta-analysis [13], observational studies have demonstrated that human milk intake is associated with less sepsis [14-17].

Long-term impact of human milk in neonatal care

Breast-fed infants have generally higher IQ scores and lower risk of obesity at school age compared to formula-fed [18]. Observational studies have suggested beneficial effects of human milk in preterm infants on health outcomes at school age, including reduced LDL/HDL cholesterol ratio, diastolic blood pressure, insulin resistance and likelihood of obesity/overweight in adolescence [19-22], higher bone mass in adolescence [23], higher cognitive performance in childhood [24, 25] and increased brain size and white matter in adolescence [26].

Nutritional inadequacy of breast milk for preterms: implications

It is well recognised, that despite the health benefits of human milk, it does not meet the nutritional needs of ELBW infants whose rates of somatic and brain growth far exceed that of term neonates. Poor growth in ELGA infants is associated with worse subsequent neurological development [27]. In order to meet the nutritional needs of enterally fed preterm

For peer review only - http://bmjopen.bmj.com/site/about/guidelines.xhtml

infants, protein containing fortifiers are therefore required, and these are generally cow's milk-based. Whilst cow's milk-based fortifiers are routinely used in modern neonatal care to provide adequate nutrition, there is some evidence, indicating that cow's milk-based fortifiers may have negative effects when compared to human milk-based fortifiers [28, 29].

Clinical studies on human milk-based breast milk nutrient fortifiers

Three randomised trials have examined the impact of human milk-based fortifies [29-31]. One examined the impact of replacing cow's milk-based preterm formulas and fortifiers with donor milk and a human milk-based fortifier in 207 infants [29]. Infants fed the human milk-based diet had a lower NEC rate (5.4% vs 15.9%) and lower risk of NEC surgery (1.4% vs 11.6%). A post hoc subgroup analysis of infants not receiving preterm formula (n=114) showed a 4.2-fold increased risk of NEC and a 5.1-fold increased risk of NEC surgery or death in infants receiving a cow's milk-based, as opposed to a human milk-based, fortifier [28]. In a Canadian RCT, on human milk-based vs. cow´s milk-based nutrient fortifier in 127 preterm infants with a birth weight <1250g, the infants were exclusively fed with breast milk [31]. The primary outcome, feeding tolerance, was similar in the two study groups. Although the trial was not powered to demonstrate a significant effect of severe complications such as NEC and late-onset sepsis, there was a significant reduction of retinopathy of prematurity (ROP) (1.6% vs 10.2%, p=0.04) in infants receiving the human milk-based fortifier.

Objectives

The primary objective is to evaluate if the supplementation of a human milk-based fortifier reduces the severe complications of NEC, sepsis and mortality as compared with bovine protein-based fortifier in extremely preterm infants fed only human breast milk (mother's own milk and/or donor milk). Secondary objectives are to evaluate if a human milk-based fortifier supplementation reduces other severe complications such as bronchopulmonary dysplasia (BPD), ROP and neurological impairment. Possible mechanisms underlying the effect of a human milk-based fortifier will be analysed in blood, stool, urine and breast milk samples. Health economic analyses will be made to evaluate the costs and benefits of an introduction of human milk-based fortifier in neonatal intensive care units (NICUs).

For peer review only

For peer review only - http://bmjopen.bmj.com/site/about/guidelines.xhtml

METHODS

Design of the N-forte study

This is a randomised controlled multi-centre superiority trial comparing diet supplementation with human breast milk-based nutrient fortifier and standard bovine protein-based nutrient fortifier in extremely preterm infants exclusively fed with human breast milk (protocol version 2020/vers.4, March 25, 2020).

Setting and participants

Enrolment of extremely preterm infants (born before gestational week 28+0) will be made at 7 level III NICUs in Sweden by an attending physician. The 7 level III NICUs and 17 level I-II neonatal units in their catchment area regions agreed to participate insuring the continuation in the event of a transfer from one hospital to another during the study period.

Inclusion

Inclusion and exclusion criteria are listed in **Table 1**. A written informed consent will be obtained from the legal guardians before inclusion (**supplementary material**). The allocation will be concealed before inclusion, but after randomisation the study is not blinded.

Randomisation

Infants are randomised 1:1 to receive either a human breast milk-based fortifier or a standard bovine protein-based nutrient fortifier before oral feeds have reached 100 mL/kg/day. Randomisation is based on the following stratification variables: primary enrolment site, gestational week (22+0-24+6 or 25+0-27+6), singleton/twin and gender. Twins are randomised together and therefore receive the same nutritional protocol. An adaptive randomisation scheme is used based on the method of minimisation. This includes a biased-coin randomisation scheme as needed in the adaptive scheme [32]. A web-based randomisation service centre is used: Randomize.net (Interrand Inc., Ottawa, Ontario, Canada).

Intervention

The active group will receive a human milk-based nutrient fortifier (Humavant+6®, Prolacta Bioscience, CA, USA). The nutrient content is displayed in **Supplementary table 1.** The control group will receive the standard bovine milk-based nutrient fortifier of the particular NICU including the infant. Adhering to the Swedish national guidelines, the intervention starts before the enteral feeds have reached 100 mL/kg/day. Any milk fortifiers must not be prescribed prior to inclusion. The attending physician and/or dietitian prescribes the enteral nutrition on a daily basis during the NICU stay, including the source of breast milk (mother's own milk and/or donor milk), total volume, and the desired level of fortification based on individual analyses of the protein content in the breast milk when such analyses have been done. The intervention is not blinded for the caregivers, clinical staff or study nurses. Targeted fortification will be applied at all study centres. The level of fortification is prescribed stepwise according to local guidelines in order to achieve appropriate protein intake and to ensure that intakes of all nutrients are within recommended ranges. The daily level of fortification for each infant is primarily based on protein intake and the aim is initially 4.0-4.5 g/kg/day but will gradually be decreased, when the infant is approaching term equivalent age and is showing adequate growth. Macronutrient analyses of MOM are performed weekly using an infrared breast milk analyser (Miris, Uppsala, Sweden). Breast milk analyses of donor breast milk are performed once for each batch. If the supplementation with the breast milk fortifier starts before the first analysis has been done, half a dose of

For peer review only - http://bmjopen.bmj.com/site/about/guidelines.xhtml

fortification is recommended during the first 1-3 days in both study groups. To assist in calculating the individual nutritional needs the computer-aided nutrition calculation program Nutrium® (Nutrium AB, Umeå, Sweden) is used. This will also be used in the prescription of other important supplements (*e.g.* vitamins, iron, calcium and phosphorous) when needed. Since the human milk-based fortifier, that will be used in this trial, have a relatively high content of Calcium and Phosphorous (**Supplementary table 1**), the total daily volume of enteral feeds should not exceed 170 mL/kg/d when the breast milk has been fortified with a full dose. Fat supplements can be considered if energy intake is low and growth is suboptimal. The infants receiving the human milk-based nutrient fortifier are supplemented with the human milk-based caloric fortifier Humavant CR® (**Supplementary table 1**), while the infants receiving standard bovine protein-based fortification are supplemented with the standard lipid products used at the unit. The infants in neither group must not be fed with formula during the intervention period, which ends at postmenstrual week 34+0. If protein fortification is still needed hereafter, there will be a transition period in the human milk-based group where the fortification of the breast milk is gradually substituted with standard bovine-based fortifier during a 5-day period.

Discontinuation

If it is in the best interest of the subject, based on the responsible physician's discretion, a patient may be discontinued from the study. The legal guardians also have the right to discontinue participation in the study at any time. The subject will still continue to be included in the study for clinical data collection, unless opposed by the legal guardians.

Primary and secondary outcomes

The enrolled infants are characterised with clinical data including growth, feeding intolerance, use of enteral and parenteral nutrition, treatment, antibiotics and complications collected daily in a study specific case report form (CRF) from birth until discharge from the hospital (not longer than postmenstrual week 44+0).

Primary and secondary clinical outcomes are listed in **Table 2**. The primary outcome is a composite of NEC stage II-III [33], culture-proven sepsis and mortality during the study period. For the NEC diagnosis, radiological assessment will be made blinded by independent radiologists. Biopsies from the intestine will be collected if acute surgery is performed. If surgery confirms NEC, there is no need of positive radiological findings for diagnosis. The final decision is confirmed by a blinded consensus panel review consisting of the investigators. If the NEC diagnosis is confirmed, this diagnosis will replace any previous sepsis diagnosis during the duration of the NEC episode. For the diagnosis of culture-proven sepsis a positive blood, urine or cerebrospinal fluid (CSF) culture is required. Furthermore, both clinical deterioration and a laboratory inflammatory response (white blood cell (WBC) count < 5 or $> 20 \times 10^9$ cells/L or total platelet count $< 100 \times 10^9$ cells/L or CRP > 15 mg/L) are required to fulfil the criteria of culture-proven sepsis [34]. All data on nutrition and other fluids will be entered daily in the Nutrium software (Nutrium AB, Umeå, Sweden). Associated predictor variables (covariates) are listed in **Table 3**.

A follow-up focusing on neurological development, growth and feeding problems will be performed at 2 years (± 3 months) of age (corrected) and 5.5 years (± 3 months) of age (uncorrected). Additional data from the 2- and 5.5-year follow-up will be obtained from the Swedish neonatal quality register (www.snq.se).

Stool, urine, blood and breast milk samples are collected and stored in a longitudinal manner for microbiology, metabolomic, proteomic, lipidomic and immunology analyses in order to

For peer review only - http://bmjopen.bmj.com/site/about/guidelines.xhtml

study underlying mechanisms. A separate protocol for each laboratory analysis will be created. Time points of collection of samples are displayed in **Figure 1**. Furthermore, vitamin and micronutrient blood levels will be analysed in a sample of infants from both study groups. Blood, stool, urine and breast milk samples are included in the biobank at Children and Women centre at the University Hospital in Linköping, County of Östergötland, Sweden.

Safety analyses and the Data and safety monitoring board

Study monitoring is made by Fravil Clinical Consulting, Stockholm, Sweden, which is independent from the sponsor and competing interest. Moderate and severe adverse events (SAE) until discharge will be recorded. Many adverse events are also considered as clinical outcomes and displayed in **Table 2**. Other adverse events that will be recorded are displayed in **Table 4**. The morbidity in extremely preterm infants is very high. Thus, a high incidence of severe and moderate adverse events unrelated to the study product could be expected in the participating infants. Typical severe conditions affecting preterm infants are listed in **Table 4**. The incidence of these SAE will be assessed by an independent data and safety monitoring board (DSMB) to make interim safety analyses after 50, 100 and 150 completed CRFs until discharge. If any SAE is significantly more common ($p<0.05$) in the human milk-based as compared with the standard group, the DSMB will bring in all medical data on infants affected by this specific SAE and assess the causality for the specific SAE in the affected infants. Based on this analysis, the DSMB will decide if the trial can continue or not after consultation with the coordinating principal investigator and the sponsor. The DSMB may decide that it is ethically correct to pursue if the positive effects of the active intervention outweigh the SAE. In addition, the investigator or the attending physicians at the study site are required to report any suspected unexpected severe adverse reaction (SUSAR) to the sponsor within 24 hours. The sponsor will report SUSARs to the manufacturer and the DSMB.

Data analysis and statistics

Estimated sample size and power:

The composite of NEC, culture-proven sepsis and mortality was 47% in extremely preterm infants surviving 3 days according to the Swedish neonatal quality register (www.snq.se). Since there is no well-powered trial with 100% coverage of breast milk, it is difficult to estimate the effect on NEC and sepsis from previous trials. With a conservative estimation, based on published studies [35-37], the incidence of the composite outcome is estimated to be reduced to 28.0%. With at least 101 infants in each group a reduction from 47% in the control to 28% in the active group would be detected at a 5% level of significance and with 80% power. With a dropout rate of 10% a total of 222 subjects are needed.

Due to uncertainties in the presupposed effect size, conditional power is estimated in the following sense. An evaluation of the overall rate of the primary clinical endpoint of NEC/sepsis/death will be made prior to the formal analysis in order to determine whether the trial sample size should be re-evaluated and increased in order to continue study enrolment. This evaluation will be based on the methodology suggested by Gould *et al.* [38]. An independent statistician, not associated with the study conduct will perform a sample size re-estimation when 150 infants have fulfilled the study period. The definitive sample size might be increased (never decreased) based on this interim analysis. Evidently, there is a need of a clinically relevant upper limit of the number of included infants. With a conservative estimation of a 50% NEC reduction in the human milk-based group, a reduction of the composite outcome from 47% in the control to 31% in the active group would be detected at a 5% level of significance and 80% power with 145 infants in each group. Based on this

For peer review only - http://bmjopen.bmj.com/site/about/guidelines.xhtml

estimation, the upper limit of included infants in this study is 322 allowing for an approximate 10% dropout rate.

The secondary outcome composite measure of culture-proven sepsis and NEC could be of special interest, as it is not affected by mortality rates, which legitimated a specific estimation of the number to include. The incidence of this outcome was 42% (www.snq.se) and could be estimated to be reduced to 26% based on the background data described above. With at least 142 infants in each group, a reduction from 42% to 26% in the active group would be detected at a 5% level of significance, 80% power and a dropout rate of 10%. A total of 312 infants would be needed for this outcome. A similar evaluation will also be made for this outcome after 150 infants have completed the study in order to determine whether the trial sample size should be re-evaluated and increased (to a maximum of 322 infants).

The power of the lab analyses will not be estimated in advance, but with at least 30 cases of the clinical outcome (*e.g.* NEC) we expect to have enough power to show relevant difference between groups.

The primary basis for all analyses of the clinical outcome will be the intention-to-treat paradigm. Only outcomes with an onset after the inclusion will be included in these analyses. Systematic bias due to drop-outs will be controlled with missing data analyses. Multiple imputation analyses will be performed if there will be a drop-out of >10%. Secondary per protocol analyses will also be made, including only infants receiving fortification and completing the intervention.

In case of a very strong effect of the active treatment, the study can be prematurely terminated based on decision by the sponsor and the DSMB, if the primary outcome is significantly lower (with a significance level <0.001) in the human milk-based group than in the standard fortification group in the interim analysis made after 150 infants have been included [39, 40]. If the significance level is ≥0.001 the study enrolment will continue (please see section 20).

Statistical methods:
The primary outcome in the clinical trial and other categorical variables will be analysed with chi-square test and adjustments for potential confounders will be made with logistic regression. Continuous variables will be analysed with Student´s t-test if the distribution is normal and Mann-Whitney U test if not. Repeated measures-ANOVA will be used for longitudinal data. Baseline characteristics will be summarized by means and standard deviations for continuous data and counts and percentages for categorical data. The statistical discrimination will be at significance level of 0.05. Bioinformatic tools will be applied for high-throughput data. Distributions between groups will be statistically tested using the analysis of similarities (ANOSIM). Principal component analyses (PCA) will be performed to display beta-diversity, and UniFrac to analyse differences in beta-diversity between the groups. Alpha diversity will be calculated using Shannon's diversity index. False discovery rate (FDR) correction will be made due to multiple comparisons (q<0.05).

Patient and public involvement
The Swedish patient organisation Svenska Prematurförbundet was involved in the planning of the study design.

Time frame for the study actions

For peer review only - http://bmjopen.bmj.com/site/about/guidelines.xhtml

Recruitment to the N-forte study began in 2019 and is planned to be completed during 2022. The study time points are presented in **Figure 1**.

For peer review only

For peer review only - http://bmjopen.bmj.com/site/about/guidelines.xhtml

DISCUSSION

There is still an important gap of evidence in this field, since there is no well-powered trial in extremely preterm infants comparing a human milk-based fortifier with bovine milk-based fortifier in a setting where both study groups are fed exclusively with breast milk. Such a trial is needed to provide evidence if a human milk-based fortifier is superior to a bovine-based one. Moreover, due to the introduction of active interventions for infants also born before gestational week 25 during the last decades [1], a trial in Nordic centres will include a substantial number of infants born in gestational week 22-25, a patient population that could be expected to gain the most of a diet free from bovine protein.

This is a prospective randomised controlled trial to achieve the highest level of evidence. However, it is not blinded as it would not be possible to prescribe the fortifier and prepare the breast milk in a blinded fashion. Firstly, the prescriptions are based on individually targeted fortifications on a daily basis by different physicians. It is essential that the clinician or the dietician knows which study group the individual patient belongs to, since the fortifiers are not exactly equal in nutrient content. Conventional fortifiers are powders mixed into the breast milk whereas human milk-based fortifiers are in liquid form and thereby substituting a part of the breastmilk given. Secondly, conducting centralised analyses and distribution of fortified breast milk [31], is not an option for logistical reasons as this is a multi-centre study with more than 20 participating hospitals all over Sweden.
The criteria for the primary outcomes of culture-proven sepsis and NEC are therefore objective. The radiological assessment of NEC will be made blinded by an independent radiologist. The secondary outcome ROP will be diagnosed by an independent ophthalmologist. There are, however, outcomes, that potentially could be affected by the unblinded design of the trial, such as feeding intolerance. The results for these outcomes will therefore be interpreted with great caution.

Primary endpoint will be a composite of NEC, culture-proven sepsis and death. The rationale for the composite variable is that NEC and sepsis share many pathogenic mechanisms and that the diagnosis of NEC and sepsis often is a continuum. Furthermore, mortality constitutes an intrinsic censoring effect in infants at high risk of developing severe sepsis or NEC. In parallel, it is also logical to introduce a mortality and morbidity index (MMI; composite measure requiring any of the following: death, NEC stage II-III, culture-proven sepsis, moderate to severe BPD or ROP stage III-V) as a secondary outcome as shown in **Table 2**. Only conditions occurring after the inclusion will be included for this outcome; hence, early debuting conditions like *e.g.* IVH and early-onset sepsis (< 72 hours) are not included.

For peer review only - http://bmjopen.bmj.com/site/about/guidelines.xhtml

ETHICS

Research addressing ELGA infants is crucial in order to achieve knowledge of possible causes underlying the development of severe complications and finding preventive strategies. Intervention studies are needed to refute or confirm the suggested effect of human milk-based nutrient fortification. We do not perceive any major health hazard with the study design. Human milk-based nutrient fortifiers are considered to be safe in ELGA infants and safety will be closely monitored.

A written informed consent will be obtained from legal guardians before inclusion. Infants or their families have no specific benefit of study participation besides the possible effects of the human milk-based nutrient fortifier in the active group. Besides the supplementation of the breast milk fortifier and the samples collected in the trial, the participating infants will not be treated differently compared to routine care. The potential benefit for future ELGA infants, however, could be substantial. Finding an intervention that reduces NEC, sepsis and mortality would have a major impact of the well-being in this patient population. It may also elicit beneficial effects on health economics. Only randomised clinical trials will give sufficient evidence for a general recommendation of a new treatment.

Personal information about enrolled participants will be collected, shared and maintained in accordance with the EU General Data Protection Regulation (GDPR).

Crown Princess Victoria Children´s Hospital, County of Östergötland, Linköping, Sweden, is the sponsor and owns all the information obtained in the trial together with the co-ordinating principal investigator. The study is made in collaboration with the company Prolacta Bioscience producing the human milk-based fortifier. The study, however, is investigator-initiated. There are no publication restrictions. None of the investigators have a financial interest in Prolacta Bioscience. The donating mother signs an informed consent form stating the rights and responsibilities of the donating mother as well as the payment for the provided breast milk. Only excess breast milk, produced by the donating mother, that is beyond the consumptive needs of her nursing child, is accepted.

The potential benefits are considered to outweigh the possible discomfort to the infants and their families. The study is conducted according to ICH/GCP guidelines and was approved by the regional ethical review board at Linköping University (Dnr 2018/193-31, Dnr 2018/384-32).

Acknowledgement

The authors wish to thank all participating hospitals and staff members and the Swedish patient organisation Svenska Prematurförbundet for their invaluable contributions in the planning and realisation of the study.

Authors´ contributions

Co-ordinating principle investigator, TA; Conceptualization, TA, FA, MD, AE; Formal analysis, TA. MD; Funding acquisition, TA; Methodology, TA, FA, MD, AE; Project administration (steering committee), TA, FA, GBJ, MD, AE, LN; Resources, TA, FA, MD, AE. LN; Supervision TA; Validation, TA, FA, MD, AE. LN; Writing – original draft, GBJ; Writing – review and editing, TA, FA, GBJ, MD, AE, LN.

Funding

This work is funded by grants from the Swedish Research Council (2020-01111 and 2019-01005), the Research Council for Southeast Sweden, ALF Grants, Prolacta Bioscience, CA, USA.

For peer review only - http://bmjopen.bmj.com/site/about/guidelines.xhtml

Competing interests

TA has got a grant for the present study by Prolacta Bioscience, CA, USA, but none of the investigators have any financial interest in Prolacta Bioscience.

For peer review only

For peer review only - http://bmjopen.bmj.com/site/about/guidelines.xhtml

REFERENCES

1. Norman, M., et al., *Association Between Year of Birth and 1-Year Survival Among Extremely Preterm Infants in Sweden During 2004-2007 and 2014-2016.* JAMA, 2019. **321**(12): p. 1188-1199.
2. Breastfeeding, A.A.o.P.S.o., *Breastfeeding and the use of human milk.* Pediatrics, 2012. **129**(3): p. e827-41.
3. Underwood, M.A., *Human milk for the premature infant.* Pediatr Clin North Am, 2013. **60**(1): p. 189-207.
4. Nutrition, E.C.o., et al., *Donor human milk for preterm infants: current evidence and research directions.* J Pediatr Gastroenterol Nutr, 2013. **57**(4): p. 535-42.
5. Group, E., *Incidence of and risk factors for neonatal morbidity after active perinatal care: extremely preterm infants study in Sweden (EXPRESS).* Acta Paediatr, 2010. **99**(7): p. 978-92.
6. Lin, P.W. and B.J. Stoll, *Necrotising enterocolitis.* The Lancet, 2006. **368**(9543): p. 1271-1283.
7. Nino, D.F., C.P. Sodhi, and D.J. Hackam, *Necrotizing enterocolitis: new insights into pathogenesis and mechanisms.* Nat Rev Gastroenterol Hepatol, 2016. **13**(10): p. 590-600.
8. Eaton, S., C.M. Rees, and N.J. Hall, *Current Research on the Epidemiology, Pathogenesis, and Management of Necrotizing Enterocolitis.* Neonatology, 2017. **111**(4): p. 423-430.
9. Roze, E., et al., *Functional impairments at school age of children with necrotizing enterocolitis or spontaneous intestinal perforation.* Pediatr Res, 2011. **70**(6): p. 619-25.
10. Humberg, A., et al., *Surgical necrotizing enterocolitis but not spontaneous intestinal perforation is associated with adverse neurological outcome at school age.* Sci Rep, 2020. **10**(1): p. 2373.
11. Ganapathy, V., J.W. Hay, and J.H. Kim, *Costs of necrotizing enterocolitis and cost-effectiveness of exclusively human milk-based products in feeding extremely premature infants.* Breastfeed Med, 2012. **7**(1): p. 29-37.
12. Ganapathy, V., et al., *Long term healthcare costs of infants who survived neonatal necrotizing enterocolitis: a retrospective longitudinal study among infants enrolled in Texas Medicaid.* BMC Pediatr, 2013. **13**: p. 127.
13. Quigley, M., N.D. Embleton, and W. McGuire, *Formula versus donor breast milk for feeding preterm or low birth weight infants.* Cochrane Database Syst Rev, 2019. **7**: p. CD002971.
14. Narayanan, I., et al., *Partial supplementation with expressed breast-milk for prevention of infection in low-birth-weight infants.* Lancet, 1980. **2**(8194): p. 561-3.
15. Narayanan, I., K. Prakash, and V.V. Gujral, *The value of human milk in the prevention of infection in the high-risk low-birth-weight infant.* J Pediatr, 1981. **99**(3): p. 496-8.
16. Narayanan, I., et al., *Randomised controlled trial of effect of raw and holder pasteurised human milk and of formula supplements on incidence of neonatal infection.* Lancet, 1984. **2**(8412): p. 1111-3.
17. Corpeleijn, W.E., et al., *Intake of own mother's milk during the first days of life is associated with decreased morbidity and mortality in very low birth weight infants during the first 60 days of life.* Neonatology, 2012. **102**(4): p. 276-81.

For peer review only

18. Horta, B.L., C. Loret de Mola, and C.G. Victora, *Breastfeeding and intelligence: a systematic review and meta-analysis.* Acta Paediatr, 2015. **104**(467): p. 14-9.
19. Horta, B.L., C. Loret de Mola, and C.G. Victora, *Long-term consequences of breastfeeding on cholesterol, obesity, systolic blood pressure and type 2 diabetes: a systematic review and meta-analysis.* Acta Paediatr, 2015. **104**(467): p. 30-7.
20. Singhal, A., et al., *Breastmilk feeding and lipoprotein profile in adolescents born preterm: follow-up of a prospective randomised study.* Lancet, 2004. **363**(9421): p. 1571-8.
21. Singhal, A., T.J. Cole, and A. Lucas, *Early nutrition in preterm infants and later blood pressure: two cohorts after randomised trials.* Lancet, 2001. **357**(9254): p. 413-9.
22. Singhal, A., et al., *Low nutrient intake and early growth for later insulin resistance in adolescents born preterm.* Lancet, 2003. **361**(9363): p. 1089-97.
23. Fewtrell, M.S., et al., *Early diet and peak bone mass: 20 year follow-up of a randomized trial of early diet in infants born preterm.* Bone, 2009. **45**(1): p. 142-9.
24. Lucas, A., et al., *Breast milk and subsequent intelligence quotient in children born preterm.* Lancet, 1992. **339**(8788): p. 261-4.
25. Anderson, J.W., B.M. Johnstone, and D.T. Remley, *Breast-feeding and cognitive development: a meta-analysis.* Am J Clin Nutr, 1999. **70**(4): p. 525-35.
26. Isaacs, E.B., et al., *Impact of breast milk on intelligence quotient, brain size, and white matter development.* Pediatr Res, 2010. **67**(4): p. 357-62.
27. Ehrenkranz, R.A., et al., *Growth in the neonatal intensive care unit influences neurodevelopmental and growth outcomes of extremely low birth weight infants.* Pediatrics, 2006. **117**(4): p. 1253-61.
28. Lucas, A., J. Boscardin, and S.A. Abrams, *Preterm Infants Fed Cow's Milk-Derived Fortifier Had Adverse Outcomes Despite a Base Diet of Only Mother's Own Milk.* Breastfeed Med, 2020. **15**(5): p. 297-303.
29. Sullivan, S., et al., *An exclusively human milk-based diet is associated with a lower rate of necrotizing enterocolitis than a diet of human milk and bovine milk-based products.* J Pediatr, 2010. **156**(4): p. 562-7.e1.
30. Cristofalo, E.A., et al., *Randomized trial of exclusive human milk versus preterm formula diets in extremely premature infants.* J Pediatr, 2013. **163**(6): p. 1592-1595 e1.
31. O'Connor, D.L., et al., *Nutrient enrichment of human milk with human and bovine milk-based fortifiers for infants born weighing <1250 g: a randomized clinical trial.* Am J Clin Nutr, 2018. **108**(1): p. 108-116.
32. Pocock, S.J. and R. Simon, *Sequential treatment assignment with balancing for prognostic factors in the controlled clinical trial.* Biometrics, 1975. **31**(1): p. 103-15.
33. Bell, M.J., et al., *Neonatal necrotizing enterocolitis. Therapeutic decisions based upon clinical staging.* Ann Surg, 1978. **187**(1): p. 1-7.
34. Gastmeier, P., et al., *Development of a surveillance system for nosocomial infections: the component for neonatal intensive care units in Germany.* J Hosp Infect, 2004. **57**(2): p. 126-31.
35. Hair, A.B., et al., *Beyond Necrotizing Enterocolitis Prevention: Improving Outcomes with an Exclusive Human Milk-Based Diet.* Breastfeeding medicine : the official journal of the Academy of Breastfeeding Medicine, 2016.

For peer review only - http://bmjopen.bmj.com/site/about/guidelines.xhtml

36. Sullivan, S., et al., *An exclusively human milk-based diet is associated with a lower rate of necrotizing enterocolitis than a diet of human milk and bovine milk-based products.* The Journal of pediatrics, 2010. **156**(4): p. 562-7 e1.
37. Cristofalo, E.A., et al., *Randomized Trial of Exclusive Human Milk versus Preterm Formula Diets in Extremely Premature Infants.* The Journal of pediatrics, 2013.
38. Gould, A.L., *Interim analyses for monitoring clinical trials that do not materially affect the type I error rate.* Stat Med, 1992. **11**(1): p. 55-66.
39. Pantiadosi, S., *Clinical trials: a methodological perspective, 2nd ed, p 384-386. John Wiley & sons, New Jersey, 2005.* .
40. Haybittle, J.L., *Repeated assessment of results in clinical trials of cancer treatment.* Br J Radiol, 1971. **44**(526): p. 793-7.
41. Jobe, A.H. and E. Bancalari, *Bronchopulmonary dysplasia.* Am J Respir Crit Care Med, 2001. **163**(7): p. 1723-9.
42. International Committee for the Classification of Retinopathy of, P., *The International Classification of Retinopathy of Prematurity revisited.* Arch Ophthalmol, 2005. **123**(7): p. 991-9.
43. Papile, L.A., et al., *Incidence and evolution of subependymal and intraventricular hemorrhage: a study of infants with birth weights less than 1,500 gm.* J Pediatr, 1978. **92**(4): p. 529-34.
44. de Vries, L.S., P. Eken, and L.M. Dubowitz, *The spectrum of leukomalacia using cranial ultrasound.* Behav Brain Res, 1992. **49**(1): p. 1-6.
45. Bayley, N., *Bayley Scales of Infant and Toddler Development*. 3rd ed. 2006, San Antonio, TX: Harcourt Assessment Inc.

For peer review only

For peer review only - http://bmjopen.bmj.com/site/about/guidelines.xhtml

FIGURES AND TABLES

Figure 1 Summary of the N-Forte study actions

	Neonatal period									Follow-up	
**Time point**	**Birth**	**<100 mL/kg/d**	**7±2 d**	**14±2 d**	**21±2 d**	**28±2 d**	**w34+0**	**w36+0**	**Discharge***	**2 years**	**5.5 years**
Enrolment											
Information given		X									
Elegibility screen		X									
Informed consent		X									
Allocation		X									
Perinatal data	X										
Intervention		◆—	—	—	—	—	—◆				
Feeding intolerance		◆—	—	—	—	—	—◆				
NEC, sepsis mortality		◆—	—	—	—	—	—	—	—◆		
Clinical data noted in the CRF on a daily basis	◆—	—	—	—	—	—	—	—	—◆		
Neurodevelopmental impairment										X	X
Antibiotics and concomitant medication		X	X	X	X	X	X	X	X		
Weight, length and head circumference**	X		X	X	X	X	X	X	X	X	X
Blood sample			X	X		X	w33+0-6				
Urine sample			X	X	X	X	w33+0-6				
Stool sample			X	X	X	X	w33+0-6				
Breast milk sample			X	X	X	X	w33+0-6				
Adverse events		X	X	X	X	X	X	X	X		

Week (w) refers to postmenstrual week. *Discharge or at latest postmenstrual week 44+0. **Only indicated when measurements must be made in the trial (weight is often measured every day).

For peer review only

For peer review only - http://bmjopen.bmj.com/site/about/guidelines.xhtml

**Table 1** Inclusion and exclusion criteria	
Inclusion criteria	
	· Gestational age at birth 22+0-27+6; based on prenatal ultrasonography<br>· Enteral feeds < 100 mL/kg/day at the day of randomisation<br>· Written informed consent from the legal guardians of the infant<br>· The home clinic of the infant has the logistics of maintaining the intervention until week 34+0
Exclusion criteria	
	· Lethal or complicated malformation known at the time of inclusion<br>· Chromosomal anomalies known at the time of inclusion<br>· No realistic hope for survival at the time of inclusion<br>· Gastrointestinal malformation known at the time of inclusion<br>· Abdominal surgery before the time of inclusion<br>· Participation in another intervention trial aiming at having an effect on growth, nutrition, feeding intolerance or severe complications such as NEC and sepsis<br>· Infants having nutrient fortifier or formula prior to randomisation

NEC, necrotising enterocolitis.

For peer review only

Table 2 Clinical outcomes

	**Description**	**Time Frame**
Primary clinical outcome		
Composite of NEC, culture-proven sepsis and mortality	Requires any of these three diagnoses to fulfil the criterion	A
Secondary clinical outcomes		
Feedingd intolerance and growth		
Time to reach full enteral feeds	The day of life the infant has received at least 150 mL/kg enteral feeds	A
Feeding interruption	Number of days feedings held for ≥12 hours or feeds reduced by >50% (mL/kg/d) not due to a clinical procedure or transitioning to the breast	A
Parenteral nutrition	Number of days of parental amino acid and/or lipid infusion; only days when the enteral feed <150 mL/kg/day should be included	A
Gastric aspirates	≥100% of pre-feed volume (2 hours volume if continuous); lower limit of 2 mL/kg	A
Stool frequency	Number of stools per day	A
Time to regain birth weight	First day of three success days when the infant weight is greater than birth weight	A
Weight, height and head circumference	Standard deviation score will be used for the calculations	B
Clinical variables for morbidity		
Infancy		
Mortality		A
Necrotising enterocolitis (NEC)	Bell´s stage II-III [33]; radiological assessment will be made blinded by an independent radiologist; the final decision will be confirmed by a blinded consensus panel review consisting of the investigators	A
Culture-proven sepsis	Positive blood- and/or urine- and/or CSF culture, clinical deterioration and laboratory inflammatory response [34]; further classified into early (<72 hours postpartum) or late (>72 hours postpartum) onset.	A
Composite of NEC and culture-proven sepsis	Requires any of these two diagnoses to fulfil the criterion	A
Mortality and morbidity index (MMI)	Composite measure requiring any of the following: death, NEC stage II-III, culture-proven sepsis, moderate to severe BPD or ROP stage III-V	
Spontaneous intestinal perforation (SIP)	Intestinal perforation without signs of intramural and/or portal gas and no signs of inflammation at surgery	A
Abdominal surgery		A
Suspected sepsis, not culture-proven	Clinical deterioration and laboratory inflammatory response but negative blood culture	A
Pneumonia	Pathological X-ray confirmed by a radiologist, need of increased respiratory support/oxygen and laboratory inflammatory response	A
Bronchopulmonary dysplasia (BPD)	Need of extra oxygen, high flow nasal cannula, CPAP or ventilator at w36+0 [41]	C
Retinopathy of prematurity (ROP)	Diagnosed by an independent ophthalmologist according to international classification; classified into stage I-V [42]; the diagnosis is set after w42+0	D
Intraventricular haemorrhage (IVH)	Assessed with ultrasound; classified into grade I-IV [43]	A
Periventricular leukomalacia (PVL)	Assessed with ultrasound and MRI; criteria according to de Vries [44]	A
Number of days with intensive care	Need of respirator or CPAP	A
Length of stay at the hospital	GW and day at discharge	A
Length of need of feeding tube	GW and day when the infant does not need it anymore	A
2-year follow-up		
Neurocognitive development	Bayleys III tested by psychologist [45]	E
Cerebral palsy	Tested by an an experienced paediatrician and/or physiotherapist	E
Epilepsy		E
Strabismus and/or impaired vision		E
Impaired hearing		E
Respiratory support	Need of extra oxygen and/or ventilatory support	F
Wheeze and/or asthma		G
Severe infections		H
Mortality	Including cause of death	H
Need of feeding tube		H
Extra nutritional support		H

For peer review only

For peer review only - http://bmjopen.bmj.com/site/about/guidelines.xhtml

Level of education of the parents		E
Family status		E
Day-care		E
5.5-year follow up		
Neurocognitive development	Wechsler Preschool and Primary Scale of Intelligence IV (WPPSI-IV) tested by a psychologist and Movement ABC-2 tested by a physiotherapist	I
Cerebral palsy	Tested by an an experienced paediatrician and/or physiotherapist	I
Epilepsy		I
Strabismus and/or impaired vision		I
Impaired hearing		I
Wheeze and/or asthma		I
Mortality	Including cause of death	J

Timeframe **A**, from birth until discharge (no longer than w44+0); **B**, at 7, 14, 21 and 28 days, end of intervention (≤ w34+0), w36+0, discharge (no longer than w44+0), and 2 years of age (corrected) and 5.5 years of age (uncorrected); **C**, at w36+0; **D**, from birth until w42+0; **E**, at 2 years of age (corrected); **F**, from w44+0 until 2 years of age; **G**, from birth until 2 years of age; **H**, from discharge or w44+0 (whether comes first) until 2 years of age; **I**, at 5.5 years of age (uncorrected); **J**, from 2 years until 5 years of age. Week (w) refers to postmenstrual week.

For peer review only

For peer review only - http://bmjopen.bmj.com/site/about/guidelines.xhtml

Table 3 Associated predictor variables (covariates)

1	Gender
2	Caesarean section
3	Multiple pregnancies
4	Birth weight and height
5	Small for gestational age (SGA), birth weight <2 SD
6	Maternal smoking during pregnancy
7	Preeclampsia, diagnosis by the responsible obstetrician
8	Chorioamnionitis, clinical diagnosis by the responsible obstetrician
9	Preterm premature rupture of membranes (PPROM), rupture > 1 hour before contractions started
10	Antenatal antibiotics, pertaining the period of the mother´s actual attendance at the hospital
11	Antenatal corticosteroids; the mother should have received at least 12 mg betamethasone. The corticosteroid prophylaxis is considered completed if the mother has received 2 doses at least 24 hours before delivery
12	Born at level 1-2 NICUs
13	Apgar score
14	Surfactant-administration
15	Intubation
16	Infant respiratory distress syndrome (IRDS), verified by X-ray
17	Mechanical ventilation, duration
18	Patent ductus arteriosus (PDA), requiring medical or surgical treatment
19	Antibiotics, drug, treatment period and number of days
20	Probiotics, name, treatment period and number of days
21	Opioids, drug, treatment period and number of days
22	Gastric acid inhibitors, drug, treatment period and number of days
23	Day of life when the supplementation of the study product was started
24	The amount of enteral feeds per day that the infants received when the supplementation of the study product was started
25	Number of days the infant has not received the study product
26	Intravenous lines, number of days
27	Insulin treatment, number of days
28	Postnatal corticosteroids, number of days
29	Inotropic drugs, number of days
30	Feeding regime, continuous or bolus
31	Amount of nutrient protein fortifier per day
32	Amount of fat supplement per day
33	Total amount of protein, fat, carbohydrate, energy and micronutrient per day
34	The relative amount of donor breast milk per day
35	Amount of extra enteral lipid
36	Breastfeeding, exclusive or partial

NICU, neonatal intensive care unit.

For peer review only

Table 4 Expected severe adverse events (SAE)
Severe infection such as pneumonia, sepsis or meningitis
CMV infection, upper respiratory viral infection
NEC, SIP and/or need of abdominal surgery
Respiratory distress syndrome (RDS) and BPD
Intracranial bleeding, PVL or hydrocephalus
Lung bleeding
Pneumothorax, pleural effusion
Pulmonary hypertension
Persistent ductus arteriosus (PDA)
Retinopathy of prematurity (ROP)
Death

CMV, cytomegalovirus; NEC, necrotising enterocolitis; SIP, spontaneous intestinal perforation; BPD, bronchopulmonary dysplasia; PVL, periventricular leukomalacia

For peer review only

Supplementary table 1 Nutritional content of the study product

Nutrient	Humavant+6®	Humavant CR®	Unit
Calories	143.82	262.22	kcal/100 mL
Fat	9.38	25.59	g/100 mL
Protein	5.92	0.82	g/100 mL
Carbohydrates	8.91	6.92	g/100 mL
Saturated fat	3.93	10.15	g/100 mL
Sugar	6.12	4.53	g/100 mL
Vitamins			
Vit A	46.21	114.65	µg/100 mL
Vit D	0.21	0.09	µg/100 mL
Vit K	<5.10	<4.85	µg/100 mL
Vit C	<1.02	<0.97	mg/100 mL
Vit B1, thiamine	5.92	<4.85	µg/100 mL
Vit B2, riboflavin	19.18	11.14	µg/100 mL
Vit B6, pyridoxine	<5.10	<4.85	µg/100 mL
Niacin	0.08	0.06	mg/100 mL
Folic acid	13.06	<2.43	µg/100 mL
Vit B12, cobalamin	<0.14	<0.14	µg/100 mL
Pantothenic acid	0.17	0.15	mg/100 mL
Biotin	<2.55	<3.17	µg/100 mL
Vit E, α-tocopherol	0.37	1.54	mg/100 mL
Minerals			
Salts	326.10	17.27	mg/100 mL
Sodium	130.69	6.77	mg/100 mL
Chloride	101.79	N/A	mg/100 mL
Potassium	187.17	64.83	mg/100 mL
Calcium	339.86	19.82	mg/100 mL
Phosphorus	189.20	2.83	mg/100 mL
Magnesium	21.48	0.02	mg/100 mL
Iron	0.13	0.33	mg/100 mL
Zinc	2.28	0.03	mg/100 mL
Copper	237.50	<0.03	µg/100 mL
Iodine	23.36	19.89	µg/100 mL
Selenium	9.65	2.07	µg/100 mL
Manganese	5.11	<4.03	µg/100 mL
Chromium	1.57	<0.41	µg/100 mL
Molybdenum	2.55	<1.94	µg/100 mL
Floride	<0.02	<0.02	mg/100 mL

For peer review only

Would you like to participate in the N-Forte study, in which we will compare two different protein fortifiers for breast milk?

Dear parents,
How can supplementary feeding of preterm infants be improved? We intend to investigate that in a research study in which we will compare two different protein fortifiers for breast milk. One of them is based on cow's milk and the other on human breast milk. The purpose of the study is to see whether the risk of contracting sepsis and inflammation in the intestines could be reduced if the necessary protein supplement that preterm infants need is coming from donated breast milk instead of cow's milk.

Why do we want to carry out this study?
A child that is born extremely premature is very sensitive and may suffer serious complications during the period of care. Medical development has made advancements and led to most of these children surviving and coping well.

The children's supplemental feeding and growth is an important challenge. The better we manage to handle this challenge, the better the child's health and development will be – both in the short and the long term. The aim is to give these small children the opportunity to grow just as well as they would have done inside the uterus, which is difficult. We know that breast milk is the best nutrition, including for premature infants. For that reason we always give the children a few milliliters of milk in the first 24 hours and then increase the amount progressively. After a few weeks, the child can be fed exclusively with breast milk and does not need any IV treatment. Primarily the mother's own pumped-out milk is given. If it is not enough, we give the child breast milk that has been donated to a breast milk bank, so that the child does not have to have any regular infant formula.

Children who have been born extremely premature have a larger nutritional need than children who are born following a regular term of pregnancy. Therefore additional fortifier (that contains protein, minerals, and vitamins) is added to the breast milk as routine practice in order for the children to grow as well as possible. The fortifier that is used at hospitals today is made from cow's milk, since there have not been any alternatives available.

A fortifier has now been developed that comes from human breast milk, i.e. breast milk that has been donated by other mothers, and there are some results from research that indicate that this "human" breast milk fortifier could have positive health effects. We now want to investigate whether the risk of infection and severe intestinal inflammation (NEC) is reduced if the children are given this fortifier instead of the traditional fortifier that is made out of cow's milk.

Who can take part in the study?
All children that are born in pregnancy week 22+0 to 27+6 and who do not have any serious abdominal condition, congenital disease, or malformations. The child cannot have had any protein supplement in the breast milk before entering the study.

How is the study conducted?
The child must be included in the study before we start administering additional fortifier in the breast milk, which most often occurs at 4-21 days of age, and it will be followed during the entire period of care during the newborn stage of life. We will also conduct a follow-up once your child has reached two years of age, and at 5.5 years of age. These follow-ups are made in connection with the regular follow-up that is made on all premature children.

For peer review only - http://bmjopen.bmj.com/site/about/guidelines.xhtml

The children who participate in the study are divided into two groups. In one of the groups, the children will be given a fortifier that is based on donated breast milk ($H^2MF^®$) until pregnancy week 34+0. If the child needs additional energy, a supplement of fat (ProlactCR®), which is also based on breast milk, will be given to the children in the group. In the other group, the children will be given the fortifier based on cow's milk that is the standard treatment at the department. If additional fat is needed, they will be given the supplement of fat that is the standard treatment at the department. The children will be randomized to one of the two groups so that half of the children will be given traditional fortifier and half will be given $H^2MF^®$. In that way we can compare and see whether the new fortifier is better than the current standard treatment. Apart from that, the children in both groups will be treated in exactly in the same way.

During the period of care, information about the child's supplemental nutrition, growth, treatments, and symptoms, as well as information about the mother that is important for the child's health, will be collected from the medical records and nutritional programs in order to interpret the results of the study correctly. Samples will be taken to investigate which mechanisms could be behind improved growth, intestinal function, and protection against infections. Blood samples will be taken from the child on four occasions during the period of care in connection with occasions when blood samples are being taken for other reasons. Samples of pumped-out breast milk will also be taken from the mother, as well as samples of feces and urine from the child. These samples will be frozen for future analyses. They will first be included in the care provider's biobank and then moved to biobank 516 of the Center of Pediatrics, Gynecology, and Obstetrics in Region Östergötland, Linköping. You may at any point request to have these samples destroyed without stating any reason.

Apart from the taking of samples and the study product, the children will not be treated any differently compared to children who do not participate in the study.

Is it dangerous?
The new dietary supplement ($H^2MF^®$) and the fat supplement (ProlactCR®) have been tested in several studies on extremely premature children before. There are no reports of any occurrences of an increase in the number of infections or other serious side effects. For safety's sake we will still check up on the children carefully.

Can you withdraw from the study?
Participation in the study is completely voluntary, and if you consent to participate but change your mind later on, you can withdraw your child from the study at any point in time. You do not have to tell us why. In such case, we will ask you whether we may still record information about the child's growth and health condition after the withdrawal. If you choose to terminate your child's participation in the study, it will not have any impact on your child's continued care. No remuneration will be paid for participating in the study. The patient insurance applies to your child, just as for all healthcare.

Which questions would we like to have answered?

- Could $H^2MF^®$ reduce the occurrence of serious diseases such as sepsis and inflammation in the intestines compared to cow's milk based protein fortifier?
- How long does it take before the child can handle having full meals of breast milk?
- How is the child's growth and development affected?
- Which factors could be behind the effect of $H^2MF^®$?
- Which other mechanisms lead to serious complications, poor growth and gastrointestinal function

For peer review only - http://bmjopen.bmj.com/site/about/guidelines.xhtml

What do we do with the results?

The results of the study will be presented in international scientific journals. If it turns out that $H^2MF^®$ reduces the risk of complications for prematurely born children, the intention is to offer this ~~protein~~ fortifier to all premature children. You will be informed of the results via letter.

Processing of personal data

The information will be taken from the medical records, and will only be used to answer the questions that we have specified above. They will be handled in accordance with the EU General Data Protection Regulation. The legal basis of the processing is the support of the General Data Protection Regulation for processing of data for research purposes. All data will be processed by group, and no unauthorized people will have access to it. It must be stored in a database for at least 10 years, and must be stored for longer if the scientific analyses are still ongoing. Region Östergötland, Region Uppsala, Region Västerbotten, the Västra Götaland Region and the Stockholm County Council have a joint responsibility as data controllers for all of the data in the saved data file. If needed, you can contact the data protection officer in Region Östergötland at the address Region Östergötland, Dataskyddsombud, 581 91 Linköping, or via email dataskyddsombud@regionostergotland.se. In study protocols, on sample tubes, and in the database, names and personal ID numbers will not be stated, but merely a specific code that is unique to your child. To be certain the all information is reported in a correct manner, a quality audit will be carried out. In independent person may then compare the forms that are filled out with the child's medical records. X-ray images may also be sent for an additional assessment to radiology specialist in another county council. For research purposes, researchers from other universities in Sweden and abroad may access data from the database. That also applies to the USA-based company Prolacta that produces the dietary supplement $H^2MF^®$. You have a right to gain access to the specific agreement that is required for data to be transferred to a company in the U.S. The Canadian company Interrand will also be given certain information that is needed when the help us with the randomization for which group the child should belong to (place of birth, if the child was born before pregnancy week 25+0, gender, and whether it is a twin or not). Names and personal ID numbers will not be sent to Interrand either. All people who have access to data are bound by professional secrecy. All results that are published in scientific journals will be presented at group level so that not individual child can be identified. You have the right to access the information about you that is processed in the study free of charge, and if needed have any errors amended. To ensure the quality of the results of the study, data collected in research studies cannot always be deleted upon your request, as stated in applicable legislation and regulations. Any complaints regarding the processing of personal data may be submitted to the Swedish Data Protection Authority

Should you have any further questions, or would like more information, please get in touch with one of us.

For the project,

[Name and title of supervising physician]	[Name and title of supervising nurse]
[Office location/clinic]	[Office location/clinic]
[Tel. No.]	[Tel. No.]
[Email]	[E-mail]

2019-07-25

For peer review only - http://bmjopen.bmj.com/site/about/guidelines.xhtml

Consent to participation

The signing legal guardians hereby consent to our child participating in the N-Forte study, which investigates whether the breast milk based protein supplement H²MF® reduces the risk of serious complications and improves feeding and growth in extremely premature children compared to cow's milk based protein supplements. Our child will either be given H²MF® or the cow's milk based protein supplement that is the standard treatment at the department.

We have read the written information. We have had the opportunity to ask questions and have had them answered to satisfaction.

We approve that an external person may review the study documents and compare with information in the medical records.

Regarding storage of biological samples
We have been informed and consent to biological samples (blood, breast milk, urine, and feces) being stored frozen in a biobank for future scientific use within the area that this project relates to. We can at any point request to have these samples destroyed without stating any reason.

We are aware that participation is completely **voluntary** and that we have the opportunity to withdraw from the study at any time, without having to state any reason.

………………………………………………	………………………………………………
The child's personal ID number	The child's last name and first name (if applicable)
………………………………………………	………………………………………………
Signature of legal guardian No. 1	Signature of legal guardian No. 2
………………………………………………	………………………………………………
Place and date	Place and date
………………………………………………	………………………………………………
Name in print letters	Name in print letters
………………………………………………	………………………………………………
Home telephone number cell phone	Home telephone number cell phone
………………………………………………	………………………………………………
	Place and date
………………………………………………	
Supervising physician (signature and name in print letters)	

For peer review only

STANDARD PROTOCOL ITEMS: RECOMMENDATIONS FOR INTERVENTIONAL TRIALS

SPIRIT 2013 Checklist: Recommended items to address in a clinical trial protocol and related documents*

Section/item	Item No	Description<br>*Marked in red are the relevant page references to the main document and supplementary material*
**Administrative information**		
Title	1	Descriptive title identifying the study design, population, interventions, and, if applicable, trial acronym<br>*Page 1*
Trial registration	2a	Trial identifier and registry name. If not yet registered, name of intended registry<br>*Page 2*
	2b	All items from the World Health Organization Trial Registration Data Set
Protocol version	3	Date and version identifier<br>*Page 5*
Funding	4	Sources and types of financial, material, and other support<br>*Page 10*
Roles and responsibilities	5a	Names, affiliations, and roles of protocol contributors<br>*Page 1 (names, affiliations); Page 10 (roles/contributions)*
	5b	Name and contact information for the trial sponsor<br>*Page 10*
	5c	Role of study sponsor and funders, if any, in study design; collection, management, analysis, and interpretation of data; writing of the report; and the decision to submit the report for publication, including whether they will have ultimate authority over any of these activities<br>*Page 10*
	5d	Composition, roles, and responsibilities of the coordinating centre, steering committee, endpoint adjudication committee, data management team, and other individuals or groups overseeing the trial, if applicable (see Item 21a for data monitoring committee)<br>*Page 10*
**Introduction**		

For peer review only - http://bmjopen.bmj.com/site/about/guidelines.xhtml

Background and rationale	6a	Description of research question and justification for undertaking the trial, including summary of relevant studies (published and unpublished) examining benefits and harms for each intervention<br>*Pages 3 to 4*
	6b	Explanation for choice of comparators<br>*Page 9*
Objectives	7	Specific objectives or hypotheses<br>*Page 4*
Trial design	8	Description of trial design including type of trial (eg, parallel group, crossover, factorial, single group), allocation ratio, and framework (eg, superiority, equivalence, noninferiority, exploratory)<br>*Page 5*

Methods: Participants, interventions, and outcomes

Study setting	9	Description of study settings (eg, community clinic, academic hospital) and list of countries where data will be collected. Reference to where list of study sites can be obtained<br>*Page 5*
Eligibility criteria	10	Inclusion and exclusion criteria for participants. If applicable, eligibility criteria for study centres and individuals who will perform the interventions (eg, surgeons, psychotherapists)<br>*Pages 5 and 16 (Table 1)*
Interventions	11a	Interventions for each group with sufficient detail to allow replication, including how and when they will be administered<br>*Pages 5 to 6 and Supplementary table 1*
	11b	Criteria for discontinuing or modifying allocated interventions for a given trial participant (eg, drug dose change in response to harms, participant request, or improving/worsening disease)<br>*Page 6*
	11c	Strategies to improve adherence to intervention protocols, and any procedures for monitoring adherence (eg, drug tablet return, laboratory tests)<br>*N/A*
	11d	Relevant concomitant care and interventions that are permitted or prohibited during the trial<br>*Page 6 ("The infants in neither group must not be fed with formula during the intervention period")*

For peer review only - http://bmjopen.bmj.com/site/about/guidelines.xhtml

Outcomes	12	Primary, secondary, and other outcomes, including the specific measurement variable (eg, systolic blood pressure), analysis metric (eg, change from baseline, final value, time to event), method of aggregation (eg, median, proportion), and time point for each outcome. Explanation of the clinical relevance of chosen efficacy and harm outcomes is strongly recommended<br>*Pages 6 to 7, 15 (Figure 1), 17 to 18 (Table 2), and 19 (Table 3)*
Participant timeline	13	Time schedule of enrolment, interventions (including any run-ins and washouts), assessments, and visits for participants. A schematic diagram is highly recommended (see Figure)<br>*Pages 5 to 6 and 15 (Figure 1)*
Sample size	14	Estimated number of participants needed to achieve study objectives and how it was determined, including clinical and statistical assumptions supporting any sample size calculations<br>*Pages 7 to 8*
Recruitment	15	Strategies for achieving adequate participant enrolment to reach target sample size<br>*Page 5 (Setting and participants)*

Methods: Assignment of interventions (for controlled trials)

Allocation:

Sequence generation	16a	Method of generating the allocation sequence (eg, computer-generated random numbers), and list of any factors for stratification. To reduce predictability of a random sequence, details of any planned restriction (eg, blocking) should be provided in a separate document that is unavailable to those who enrol participants or assign interventions<br>*Page 5*
Allocation concealment mechanism	16b	Mechanism of implementing the allocation sequence (eg, central telephone; sequentially numbered, opaque, sealed envelopes), describing any steps to conceal the sequence until interventions are assigned<br>*Page 5*
Implementation	16c	Who will generate the allocation sequence, who will enrol participants, and who will assign participants to interventions<br>*Page 5*
Blinding (masking)	17a	Who will be blinded after assignment to interventions (eg, trial participants, care providers, outcome assessors, data analysts), and how<br>*Page 9 ("The radiological assessment of NEC will be made blinded by an independent radiologist. The secondary outcome ROP will be diagnosed by an independent ophthalmologist.")*

	17b	If blinded, circumstances under which unblinding is permissible, and procedure for revealing a participant's allocated intervention during the trial<br>*N/A*

Methods: Data collection, management, and analysis

Data collection methods	18a	Plans for assessment and collection of outcome, baseline, and other trial data, including any related processes to promote data quality (eg, duplicate measurements, training of assessors) and a description of study instruments (eg, questionnaires, laboratory tests) along with their reliability and validity, if known. Reference to where data collection forms can be found, if not in the protocol<br>*Pages 6 to 7*
	18b	Plans to promote participant retention and complete follow-up, including list of any outcome data to be collected for participants who discontinue or deviate from intervention protocols<br>*Page 6*
Data management	19	Plans for data entry, coding, security, and storage, including any related processes to promote data quality (eg, double data entry; range checks for data values). Reference to where details of data management procedures can be found, if not in the protocol<br>*Page 7*
Statistical methods	20a	Statistical methods for analysing primary and secondary outcomes. Reference to where other details of the statistical analysis plan can be found, if not in the protocol<br>*Page 8*
	20b	Methods for any additional analyses (eg, subgroup and adjusted analyses)<br>*Page 8*
	20c	Definition of analysis population relating to protocol non-adherence (eg, as randomised analysis), and any statistical methods to handle missing data (eg, multiple imputation)<br>*N/A*

Methods: Monitoring

Data monitoring	21a	Composition of data monitoring committee (DMC); summary of its role and reporting structure; statement of whether it is independent from the sponsor and competing interests; and reference to where further details about its charter can be found, if not in the protocol. Alternatively, an explanation of why a DMC is not needed<br>*Page 7*

	21b	Description of any interim analyses and stopping guidelines, including who will have access to these interim results and make the final decision to terminate the trial<br>*Pages 7 to 8*
Harms	22	Plans for collecting, assessing, reporting, and managing solicited and spontaneously reported adverse events and other unintended effects of trial interventions or trial conduct<br>*Pages 7 and 20 (Table 4)*
Auditing	23	Frequency and procedures for auditing trial conduct, if any, and whether the process will be independent from investigators and the sponsor<br>*Page 7*
**Ethics and dissemination**		
Research ethics approval	24	Plans for seeking research ethics committee/institutional review board (REC/IRB) approval<br>*N/A (Already approved by the regional ethical review board in Linköping Sweden (Dnr 2018/193-31, Dnr 2018/384-32)*
Protocol amendments	25	Plans for communicating important protocol modifications (eg, changes to eligibility criteria, outcomes, analyses) to relevant parties (eg, investigators, REC/IRBs, trial participants, trial registries, journals, regulators)<br>*N/A*
Consent or assent	26a	Who will obtain informed consent or assent from potential trial participants or authorised surrogates, and how (see Item 32)<br>*Page 5*
	26b	Additional consent provisions for collection and use of participant data and biological specimens in ancillary studies, if applicable<br>*Supplementary material (Parent Information and consent)*
Confidentiality	27	How personal information about potential and enrolled participants will be collected, shared, and maintained in order to protect confidentiality before, during, and after the trial<br>*Supplementary material (Parent Information and consent)*
Declaration of interests	28	Financial and other competing interests for principal investigators for the overall trial and each study site<br>*Page 11*
Access to data	29	Statement of who will have access to the final trial dataset, and disclosure of contractual agreements that limit such access for investigators<br>*Page 10*

For peer review only - http://bmjopen.bmj.com/site/about/guidelines.xhtml

Ancillary and post-trial care	30	Provisions, if any, for ancillary and post-trial care, and for compensation to those who suffer harm from trial participation<br>*N/A*
Dissemination policy	31a	Plans for investigators and sponsor to communicate trial results to participants, healthcare professionals, the public, and other relevant groups (eg, via publication, reporting in results databases, or other data sharing arrangements), including any publication restrictions<br>*Pages 2, 10, and supplementary material (Parent Information and consent)*
	31b	Authorship eligibility guidelines and any intended use of professional writers<br>*Page 10*
	31c	Plans, if any, for granting public access to the full protocol, participant-level dataset, and statistical code<br>*N/A*
**Appendices**		
Informed consent materials	32	Model consent form and other related documentation given to participants and authorised surrogates<br>*Supplementary material (Parent Information and consent)*
Biological specimens	33	Plans for collection, laboratory evaluation, and storage of biological specimens for genetic or molecular analysis in the current trial and for future use in ancillary studies, if applicable<br>*Page 7 and supplementary material (Parent Information and consent)*

*It is strongly recommended that this checklist be read in conjunction with the SPIRIT 2013 Explanation & Elaboration for important clarification on the items. Amendments to the protocol should be tracked and dated. The SPIRIT checklist is copyrighted by the SPIRIT Group under the Creative Commons "Attribution-NonCommercial-NoDerivs 3.0 Unported" license.

For peer review only

BMJ Open

Nordic study on human milk fortification in extremely preterm infants (N forte): the study protocol of a randomised controlled trial

Journal:	*BMJ Open*
Manuscript ID	bmjopen-2021-053400.R1
Article Type:	Protocol
Date Submitted by the Author:	29-Sep-2021
Complete List of Authors:	Jensen, Georg Bach; Linköping University, Department of Biomedical and Clinical Sciences; Linköping University, Department of Paediatrics<br>Ahlsson, Fredrik ; Uppsala University, Department of Women´s and Children´s Health<br>Domellöf, Magnus; Umea University, Department of Clinical Sciences, Pediatrics<br>Elfvin, Anders; Sahlgrenska Academy, Institute of Clinical Sciences, Department of Paediatrics; Sahlgrenska University Hospital, Queen Silvia Children's Hospital, Department of Paediatrics<br>Naver, Lars; Karolinska Institute, Department of Clinical Science, Intervention, and Technology; Karolinska University Hospital, Department of Neonatal Medicine<br>Abrahamsson, Thomas; Linköping University, Department of Biomedical and Clinical Sciences; Linköping University, Department of Paediatrics
**Primary Subject Heading**:	Paediatrics
Secondary Subject Heading:	Nutrition and metabolism
Keywords:	Neonatal intensive & critical care < INTENSIVE & CRITICAL CARE, NEONATOLOGY, Developmental neurology & neurodisability < PAEDIATRICS, NUTRITION & DIETETICS

SCHOLARONE™
Manuscripts

I, the Submitting Author has the right to grant and does grant on behalf of all authors of the Work (as defined in the below author licence), an exclusive licence and/or a non-exclusive licence for contributions from authors who are: i) UK Crown employees; ii) where BMJ has agreed a CC-BY licence shall apply, and/or iii) in accordance with the terms applicable for US Federal Government officers or employees acting as part of their official duties; on a worldwide, perpetual, irrevocable, royalty-free basis to BMJ Publishing Group Ltd ("BMJ") its licensees and where the relevant Journal is co-owned by BMJ to the co-owners of the Journal, to publish the Work in this journal and any other BMJ products and to exploit all rights, as set out in our licence.

The Submitting Author accepts and understands that any supply made under these terms is made by BMJ to the Submitting Author unless you are acting as an employee on behalf of your employer or a postgraduate student of an affiliated institution which is paying any applicable article publishing charge ("APC") for Open Access articles. Where the Submitting Author wishes to make the Work available on an Open Access basis (and intends to pay the relevant APC), the terms of reuse of such Open Access shall be governed by a Creative Commons licence – details of these licences and which Creative Commons licence will apply to this Work are set out in our licence referred to above.

Other than as permitted in any relevant BMJ Author's Self Archiving Policies, I confirm this Work has not been accepted for publication elsewhere, is not being considered for publication elsewhere and does not duplicate material already published. I confirm all authors consent to publication of this Work and authorise the granting of this licence.

For peer review only

Nordic study on human milk fortification in extremely preterm infants: a randomised controlled trial: the N-forte trial

Georg Bach Jensen[1], Fredrik Ahlsson[2], Magnus Domellöf[3], Anders Elfvin[4,5], Lars Navér[6,7], Thomas Abrahamsson[1]

[1] Department of Biomedical and Clinical Sciences and Department of Paediatrics, Linköping University, Linköping, Sweden

[2] Department of Women´s and Children´s Health, Uppsala University, Uppsala, Sweden

[3] Department of Clinical Science, Pediatrics, Umeå University, Umeå, Sweden

[4] Department of Paediatrics, Institute of Clinical Sciences, Sahlgrenska Academy, University of Gothenburg, Gothenburg, Sweden

[5] Region Västra Götaland, Sahlgrenska University Hospital, Queen Silvia Children's Hospital, Department of Paediatrics, Gothenburg, Sweden

[6] Department of Clinical Science, Intervention, and Technology, Karolinska Institutet, Stockholm, Sweden

[7] Department of Neonatal Medicine, Karolinska University Hospital, Stockholm, Sweden

Corresponding author:
Thomas Abrahamsson
Department of Biomedical and Clinical Sciences and Department of Paediatrics, Linköping University,
Crown Princess Victoria Children´s hospital
University Hospital
SE-581 85 Linköping
Sweden
thomas.abrahamsson@liu.se
+46-709566815

For peer review only - http://bmjopen.bmj.com/site/about/guidelines.xhtml

ABSTRACT

Introduction

The mortality rate of extremely low gestational age (ELGA) (born < gestational week 28+0) infants remains high, and severe infections and necrotising enterocolitis (NEC) are common causes of death. Preterm infants receiving human milk have lower incidence of sepsis and NEC than those fed a bovine milk-based preterm formula. Despite this, fully human milk fed ELGA infants most often have a significant intake of cow's milk protein from bovine-based protein fortifier. The aim of this study is to evaluate whether the supplementation of human milk-based, as compared with bovine-based, nutrient fortifier reduces the prevalence of NEC, sepsis and mortality in ELGA infants exclusively fed with human milk.

Methods and analysis

A randomised-controlled multi-centre trial comparing the effect of a human breast milk-based fortifier with a standard bovine protein-based fortifier in 222 to 322 ELGA infants fed human breast milk (mother´s own milk and/or donor milk). The infants will be randomised to either fortifier before reaching 100 mL/kg/day in oral feeds. The intervention, stratified by centre, will continue until the target postmenstrual week 34+0. The primary outcome is a composite of NEC, sepsis or death. Infants are characterised with comprehensive clinical and nutritional data collected prospectively from birth until hospital discharge. Stool, urine, blood and breast milk samples are collected for analyses in order to study underlying mechanisms. A follow-up focusing on neurological development and growth will be performed at 2 and 5.5 years of age. Health economic analyses will be made.

Ethics and dissemination

The study is conducted according to ICH/GCP guidelines and is approved by the regional ethical review board in Linköping Sweden (Dnr 2018/193-31, Dnr 2018/384-32). Results will be presented at scientific meetings and published in peer-reviewed publications.

Trial registration number

The study was registered with ClinicalTrials.gov NCT03797157, January 9th, 2019.

STRENGTHS AND LIMITATIONS OF THIS STUDY

- A randomised-controlled multi-centre trial on human milk-based fortification in ELGA infants powered to detect a reduction in sepsis, NEC and mortality as a composite outcome.
- Several assessments of outcomes will be made blinded, such as the radiological assessment of NEC.
- Both study groups will receive exclusively enteral feeding with breast milk and thereby not differ, besides the different breast milk fortification.
- A significant positive result of this trial would justify implementation of human milk-based fortifiers in the neonatal intensive care units.
- Targeted fortification of breast milk and the difference in nutrient content between the study product and the standard fortification made it impossible to blind the study after randomisation.

For peer review only - http://bmjopen.bmj.com/site/about/guidelines.xhtml

INTRODUCTION

Extremely preterm infants are at risk of severe morbidity and mortality

Although neonatal care has improved markedly during the last decades, mortality and morbidity remain high with a 1-year mortality of 23% in ELGA neonates in affluent countries [1]. It is well known that nutrition is a key factor for survival, clinical course and later outcome of high-risk preterm infants; and of particular relevance to this trial is the evidence for beneficial roles of human milk in neonatal care, underpinning current recommendations for its use [2-4]. Extremely preterm infants (born before 28 weeks of completed gestation) are initially fed both enterally and parenterally while enteral feed tolerance develops. The primary choice is the infant's own mother's milk. Preterm formula or donor breast milk can be used when mothers provide insufficient amounts of breast milk, and in the Nordic countries, donor breast milk is routinely used for preterm infants up to 32-34 weeks postmenstrual age. A growing literature supports the use of human milk for feeding preterm infants, in particular for the smallest and most vulnerable preterm infants.

Reduction of necrotising enterocolitis (NEC)

NEC remains a significant problem in neonates with an incidence between 4 to 15% in very low birth weight (VLBW; <1500g) infants [1, 5, 6]. The pathogenesis is multifactorial, and the disease leads to intestinal inflammation, ischemia and necrosis. The main risk factors besides prematurity, are formula feeding, hypoxic–ischaemic injury, and abnormal bacterial gut colonisation [7, 8]. NEC frequently results in death (around 20-30%) or need for surgical resection of necrotic gut which may lead to short bowel syndrome. Further, NEC is a major adverse factor for neurological impairment [9, 10]. The health economic cost of NEC is large [11, 12]. The most recent Cochrane meta-analysis, including 8 randomized controlled trials with a total of 1605 preterm infants, shows that human milk, as compared to formula, reduces the risk of NEC by a factor 1.9 but it is not clear whether this effect is due to protective factors in human milk or the detrimental effects of cow's milk protein in formula [13].

Reduction in neonatal sepsis

Neonatal late onset sepsis remains a life-threatening and common problem in neonatal care. The incidence is 35-40% in Swedish ELGA infants (www.snq.se). Growing evidence links sepsis to the mode of feeding. Even though no significant effect of human milk on sepsis was shown in the latest meta-analysis [13], observational studies have demonstrated that human milk intake is associated with less sepsis [14-17].

Long-term impact of human milk in neonatal care

Breast-fed infants have generally higher IQ scores and lower risk of obesity at school age compared to formula-fed [18]. Observational studies have suggested beneficial effects of human milk in preterm infants on health outcomes at school age, including reduced LDL/HDL cholesterol ratio, diastolic blood pressure, insulin resistance and likelihood of obesity/overweight in adolescence [19-22], higher bone mass in adolescence [23], higher cognitive performance in childhood [24, 25] and increased brain size and white matter in adolescence [26].

Nutritional inadequacy of breast milk for preterms: implications

It is well recognised, that despite the health benefits of human milk, it does not meet the nutritional needs of ELBW infants whose rates of somatic and brain growth far exceed that of term neonates. Poor growth in ELGA infants is associated with worse subsequent neurological development [27]. In order to meet the nutritional needs of enterally fed preterm

For peer review only - http://bmjopen.bmj.com/site/about/guidelines.xhtml

infants, protein containing fortifiers are therefore required, and these are generally cow's milk-based. Whilst cow's milk-based fortifiers are routinely used in modern neonatal care to provide adequate nutrition, there is some evidence, indicating that cow's milk-based fortifiers may have negative effects when compared to human milk-based fortifiers [28, 29].

Clinical studies on human milk-based breast milk nutrient fortifiers

Three randomised trials have examined the impact of human milk-based vs. bovine-based diets [29-31] of which only one study was truly designed to evaluate the impact of a human milk-based fortifier [31]. One examined the impact of replacing cow's milk-based preterm formulas and fortifiers with donor milk and a human milk-based fortifier in 207 infants [29]. Infants fed the human milk-based diet had a lower NEC rate (5.4% vs 15.9%) and lower risk of NEC surgery (1.4% vs 11.6%). A post hoc subgroup analysis of infants not receiving preterm formula (n=114) showed a 4.2-fold increased risk of NEC and a 5.1-fold increased risk of NEC surgery or death in infants receiving a cow's milk-based, as opposed to a human milk-based, fortifier [28]. In a Canadian RCT, on human milk-based vs. cow´s milk-based nutrient fortifier in 127 preterm infants with a birth weight <1250g, the infants were exclusively fed with breast milk [31]. The primary outcome, feeding tolerance, was similar in the two study groups. Although the trial was not powered to demonstrate a significant effect of severe complications such as NEC and late-onset sepsis, there was a significant reduction of retinopathy of prematurity (ROP) (1.6% vs 10.2%, p=0.04) in infants receiving the human milk-based fortifier.

Objectives

The primary objective is to evaluate if the supplementation of a human milk-based fortifier reduces the severe complications of NEC, sepsis and mortality as compared with bovine protein-based fortifier in extremely preterm infants fed only human breast milk (mother's own milk and/or donor milk). Secondary objectives are to evaluate if a human milk-based fortifier supplementation reduces other severe complications such as bronchopulmonary dysplasia (BPD), ROP and neurological impairment. Possible mechanisms underlying the effect of a human milk-based fortifier will be analysed in blood, stool, urine and breast milk samples. Health economic analyses will be made to evaluate the costs and benefits of an introduction of human milk-based fortifier in neonatal intensive care units (NICUs).

For peer review only

METHODS

Design of the N-forte study

This is a randomised controlled multi-centre superiority trial comparing diet supplementation with human breast milk-based nutrient fortifier and standard bovine protein-based nutrient fortifier in extremely preterm infants exclusively fed with human breast milk (protocol version 2020/vers.4, March 25, 2020).

Setting and participants

Enrolment of extremely preterm infants (born before gestational week 28+0) will be made at 7 level III NICUs in Sweden by an attending physician. The 7 level III NICUs and 17 level I-II neonatal units in their catchment area regions agreed to participate insuring the continuation in the event of a transfer from one hospital to another during the study period.

Inclusion

Inclusion and exclusion criteria are listed in **Table 1**. A written informed consent will be obtained from the legal guardians before inclusion (**supplementary material**). The allocation will be concealed before inclusion, but after randomisation the study is not blinded.

Randomisation

Infants are randomised 1:1 to receive either a human breast milk-based fortifier or a standard bovine protein-based nutrient fortifier before oral feeds have reached 100 mL/kg/day. Randomisation is based on the following stratification variables: primary enrolment site, gestational week (22+0-24+6 or 25+0-27+6), singleton/twin and gender. Twins are randomised together and therefore receive the same nutritional protocol. An adaptive randomisation scheme is used based on the method of minimisation. This includes a biased-coin randomisation scheme as needed in the adaptive scheme [32]. A web-based randomisation service centre is used: Randomize.net (Interrand Inc., Ottawa, Ontario, Canada).

Intervention

The active group will receive a human milk-based nutrient fortifier (Humavant+6®, Prolacta Bioscience, CA, USA). The nutrient content is displayed in **Supplementary table 1.** The control group will receive the standard bovine milk-based nutrient fortifier of the particular NICU including the infant. Adhering to the Swedish national guidelines, the intervention starts before the enteral feeds have reached 100 mL/kg/day. Any milk fortifiers must not be prescribed prior to inclusion. The attending physician and/or dietitian prescribes the enteral nutrition on a daily basis during the NICU stay, including the source of breast milk (mother's own milk and/or donor milk), total volume, and the desired level of fortification based on individual analyses of the true protein content in the breast milk when such analyses have been done. The intervention is not blinded for the caregivers, clinical staff or study nurses. Targeted fortification will be applied at all study centres. The level of fortification is prescribed stepwise according to local guidelines in order to achieve appropriate protein intake and to ensure that intakes of all nutrients are within recommended ranges. The daily level of fortification for each infant is primarily based on protein intake and the aim is initially 4.0-4.5 g/kg/day but will gradually be decreased, when the infant is approaching term equivalent age and is showing adequate growth. The growth will be assessed by the physicians in charge on a daily basis guided by the same growth charts that are used by all participating NICUs and according to Swedish national guidelines. Macronutrient analyses of MOM are performed weekly using an infrared breast milk analyser (Miris, Uppsala, Sweden).

For peer review only - http://bmjopen.bmj.com/site/about/guidelines.xhtml

Breast milk analyses of donor breast milk are performed once for each batch. If the supplementation with the breast milk fortifier starts before the first analysis has been done, half a dose of fortification is recommended during the first 1-3 days in both study groups. To assist in calculating the individual nutritional needs the computer-aided nutrition calculation program Nutrium® (Nutrium AB, Umeå, Sweden) is used. This will also be used in the prescription of other important supplements (*e.g.* vitamins, iron, calcium and phosphorous) when needed. Since the human milk-based fortifier, that will be used in this trial, have a relatively high content of Calcium and Phosphorous (**Supplementary table 1**), the total daily volume of enteral feeds should not exceed 170 mL/kg/d when the breast milk has been fortified with a full dose. Fat supplements can be considered if energy intake is low and growth is suboptimal. The infants receiving the human milk-based nutrient fortifier are supplemented with the human milk-based caloric fortifier Humavant CR® (**Supplementary table 1**), while the infants receiving standard bovine protein-based fortification are supplemented with the standard lipid products used at the unit. The infants in both groups must not be fed with formula during the intervention period, which ends at postmenstrual week 34+0. If protein fortification is still needed hereafter, there will be a transition period in the human milk-based group where the fortification of the breast milk is gradually substituted with standard bovine-based fortifier during a 5-day period.

Discontinuation

If it is in the best interest of the subject, based on the responsible physician's discretion, a patient may be discontinued from the study. The legal guardians also have the right to discontinue participation in the study at any time. The subject will still continue to be included in the study for clinical data collection, unless opposed by the legal guardians.

Primary and secondary outcomes

The enrolled infants are characterised with clinical data including growth, feeding intolerance, use of enteral and parenteral nutrition, treatment, antibiotics and complications collected daily in a study specific case report form (CRF) from birth until discharge from the hospital (not longer than postmenstrual week 44+0).

Primary and secondary clinical outcomes are listed in **Table 2**. The primary outcome is a composite of NEC stage II-III [33], culture-proven sepsis and mortality during the study period. For the NEC diagnosis, radiological assessment will be made blinded by independent radiologists. Biopsies from the intestine will be collected if acute surgery is performed. If surgery confirms NEC, there is no need of positive radiological findings for diagnosis. The final decision is confirmed by a blinded consensus panel review consisting of the investigators. If the NEC diagnosis is confirmed, this diagnosis will replace any previous sepsis diagnosis during the duration of the NEC episode. For the diagnosis of culture-proven sepsis a positive blood, urine or cerebrospinal fluid (CSF) culture is required. Furthermore, both clinical deterioration and a laboratory inflammatory response (white blood cell (WBC) count < 5 or > 20 x10^9 cells/L or total platelet count < 100 x10^9 cells/L or CRP > 15 mg/L) are required to fulfil the criteria of culture-proven sepsis [34]. All data on nutrition and other fluids will be entered daily in the Nutrium software (Nutrium AB, Umeå, Sweden). Associated predictor variables (covariates) are listed in **Table 3**.

A follow-up focusing on neurological development, growth and feeding problems will be performed at 2 years (± 3 months) of age (corrected) and 5.5 years (± 3 months) of age (uncorrected). Additional data from the 2- and 5.5-year follow-up will be obtained from the Swedish neonatal quality register (www.snq.se).

For peer review only - http://bmjopen.bmj.com/site/about/guidelines.xhtml

Stool, urine, blood and breast milk samples are collected and stored in a longitudinal manner for microbiology, metabolomic, proteomic, lipidomic and immunology analyses in order to study underlying mechanisms. A separate protocol for each laboratory analysis will be created. Time points of collection of samples are displayed in **Figure 1**. Furthermore, vitamin and micronutrient blood levels will be analysed in a sample of infants from both study groups. Blood, stool, urine and breast milk samples are included in the biobank at Children and Women centre at the University Hospital in Linköping, County of Östergötland, Sweden.

Safety analyses and the Data and safety monitoring board

Study monitoring is made by Fravil Clinical Consulting, Stockholm, Sweden, which is independent from the sponsor and competing interest. Moderate and severe adverse events (SAE) until discharge will be recorded. Many adverse events are also considered as clinical outcomes and displayed in **Table 2**. Other adverse events that will be recorded are displayed in **Table 4**. The morbidity in extremely preterm infants is very high. Thus, a high incidence of severe and moderate adverse events unrelated to the study product could be expected in the participating infants. Typical severe conditions affecting preterm infants are listed in **Table 4**. The incidence of these SAE will be assessed by an independent data and safety monitoring board (DSMB) to make interim safety analyses after 50, 100 and 150 completed CRFs until discharge. If any SAE is significantly more common ($p<0.05$) in the human milk-based as compared with the standard group, the DSMB will bring in all medical data on infants affected by this specific SAE and assess the causality for the specific SAE in the affected infants. Based on this analysis, the DSMB will decide if the trial can continue or not after consultation with the coordinating principal investigator and the sponsor. The DSMB may decide that it is ethically correct to pursue if the positive effects of the active intervention outweigh the SAE. In addition, the investigator or the attending physicians at the study site are required to report any suspected unexpected severe adverse reaction (SUSAR) to the sponsor within 24 hours. The sponsor will report SUSARs to the manufacturer and the DSMB.

Data analysis and statistics

Estimated sample size and power:

The composite of NEC, culture-proven sepsis and mortality was 47% in extremely preterm infants surviving 3 days according to the Swedish neonatal quality register (www.snq.se). Since there is no well-powered trial with 100% coverage of breast milk, it is difficult to estimate the effect on NEC and sepsis from previous trials. With a conservative estimation, based on published studies [35-37], the incidence of the composite outcome is estimated to be reduced to 28.0%. With at least 101 infants in each group a reduction from 47% in the control to 28% in the active group would be detected at a 5% level of significance and with 80% power. With a dropout rate of 10% a total of 222 subjects are needed.

Due to uncertainties in the presupposed effect size, conditional power is estimated in the following sense. An evaluation of the overall rate of the primary clinical endpoint of NEC/sepsis/death will be made prior to the formal analysis in order to determine whether the trial sample size should be re-evaluated and increased in order to continue study enrolment. This evaluation will be based on the methodology suggested by Gould *et al.* [38]. An independent statistician, not associated with the study conduct will perform a sample size re-estimation when 150 infants have fulfilled the study period. The definitive sample size might be increased (never decreased) based on this interim analysis. Evidently, there is a need of a clinically relevant upper limit of the number of included infants. With a conservative estimation of a 50% NEC reduction in the human milk-based group, a reduction of the

For peer review only - http://bmjopen.bmj.com/site/about/guidelines.xhtml

composite outcome from 47% in the control to 31% in the active group would be detected at a 5% level of significance and 80% power with 145 infants in each group. Based on this estimation, the upper limit of included infants in this study is 322 allowing for an approximate 10% dropout rate.

The secondary outcome composite measure of culture-proven sepsis and NEC could be of special interest, as it is not affected by mortality rates, which legitimated a specific estimation of the number to include. The incidence of this outcome was 42% (www.snq.se) and could be estimated to be reduced to 26% based on the background data described above. With at least 142 infants in each group, a reduction from 42% to 26% in the active group would be detected at a 5% level of significance, 80% power and a dropout rate of 10%. A total of 312 infants would be needed for this outcome. A similar evaluation will also be made for this outcome after 150 infants have completed the study in order to determine whether the trial sample size should be re-evaluated and increased (to a maximum of 322 infants).

The power of the lab analyses will not be estimated in advance, but with at least 30 cases of the clinical outcome (*e.g.* NEC) we expect to have enough power to show relevant difference between groups.

The primary basis for all analyses of the clinical outcome will be the intention-to-treat paradigm. Only outcomes with an onset after the inclusion will be included in these analyses. Systematic bias due to drop-outs will be controlled with missing data analyses. Multiple imputation analyses will be performed if there will be a drop-out of >10%. Secondary per protocol analyses will also be made, including only infants receiving fortification and completing the intervention.

In case of a very strong effect of the active treatment, the study can be prematurely terminated based on decision by the sponsor and the DSMB, if the primary outcome is significantly lower (with a significance level <0.001) in the human milk-based group than in the standard fortification group in the interim analysis made after 150 infants have been included [39, 40]. If the significance level is ≥0.001 the study enrolment will continue (please see section 20).

Statistical methods:
The primary outcome in the clinical trial and other categorical variables will be analysed with chi-square test and adjustments for potential confounders will be made with logistic regression. Continuous variables will be analysed with Student´s t-test if the distribution is normal and Mann-Whitney U test if not. Repeated measures-ANOVA will be used for longitudinal data. Baseline characteristics will be summarized by means and standard deviations for continuous data and counts and percentages for categorical data. The statistical discrimination will be at significance level of 0.05. Bioinformatic tools will be applied for high-throughput data. Distributions between groups will be statistically tested using the analysis of similarities (ANOSIM). Principal component analyses (PCA) will be performed to display beta-diversity, and UniFrac to analyse differences in beta-diversity between the groups. Alpha diversity will be calculated using Shannon's diversity index. False discovery rate (FDR) correction will be made due to multiple comparisons (q<0.05).

Patient and public involvement
The Swedish patient organisation Svenska Prematurförbundet was involved in the planning of the study design.

For peer review only - http://bmjopen.bmj.com/site/about/guidelines.xhtml

For peer review only

Time frame for the study actions

Recruitment to the N-forte study began in 2019 and is planned to be completed during 2022. The study time points are presented in **Figure 1**.

For peer review only

For peer review only - http://bmjopen.bmj.com/site/about/guidelines.xhtml

DISCUSSION

There is still an important gap of evidence in this field, since there is no well-powered trial in extremely preterm infants comparing a human milk-based fortifier with bovine milk-based fortifier in a setting where both study groups are fed exclusively with breast milk. Such a trial is needed to provide evidence if a human milk-based fortifier is superior to a bovine-based one. Moreover, due to the introduction of active interventions for infants also born before gestational week 25 during the last decades [1], a trial in Nordic centres will include a substantial number of infants born in gestational week 22-25, a patient population that could be expected to gain the most of a diet free from bovine protein.

This is a prospective randomised controlled trial to achieve the highest level of evidence. However, it is not blinded as it would not be possible to prescribe the fortifier and prepare the breast milk in a blinded fashion. Firstly, the prescriptions are based on individually targeted fortifications on a daily basis by different physicians. It is essential that the clinician or the dietician knows which study group the individual patient belongs to, since the fortifiers are not exactly equal in nutrient content. Conventional fortifiers are powders mixed into the breast milk whereas human milk-based fortifiers are in liquid form and thereby substituting a part of the breastmilk given. Secondly, conducting centralised analyses and distribution of fortified breast milk [31], is not an option for logistical reasons as this is a multi-centre study with more than 20 participating hospitals all over Sweden.
The criteria for the primary outcomes of culture-proven sepsis and NEC are therefore objective. The radiological assessment of NEC will be made blinded by an independent radiologist. The secondary outcome ROP will be diagnosed by an independent ophthalmologist. There are, however, outcomes, that potentially could be affected by the unblinded design of the trial, such as feeding intolerance. The results for these outcomes will therefore be interpreted with great caution.

Primary endpoint will be a composite of NEC, culture-proven sepsis and death. The rationale for the composite variable is that NEC and sepsis share many pathogenic mechanisms and that the diagnosis of NEC and sepsis often is a continuum. Furthermore, mortality constitutes an intrinsic censoring effect in infants at high risk of developing severe sepsis or NEC. In parallel, it is also logical to introduce a mortality and morbidity index (MMI; composite measure requiring any of the following: death, NEC stage II-III, culture-proven sepsis, moderate to severe BPD or ROP stage III-V) as a secondary outcome as shown in **Table 2**. Only conditions occurring after the inclusion will be included for this outcome; hence, early debuting conditions like *e.g.* IVH (intraventricular haemorrhage) and early-onset sepsis (< 72 hours) are not included.

Macronutrient analyses of MOM are performed weekly for the targeted fortification. It has been argued that breast milk analyses should be done on a daily basis because of the day-to-day variations [41]. Such approach is not clinically feasible as it will consume far too much of MOM milk to be ethical justified. Consequently, the protein and energy intake by the neonates fed exclusively or mainly own mother's milk may be out of the target levels, while this will not apply for infants mainly fed by donor milk, since all donor milk will be analysed. These differences are not expected to have a significant impact on the primary outcome measures, especially not when the study groups are randomised, but they may affect some of the secondary outcomes, such as growth and metabolomics. However, we will have thorough data on the amount of donor milk the infant has received each day, which then will be taken in account in subsequent analyses.

For peer review only - http://bmjopen.bmj.com/site/about/guidelines.xhtml

For peer review only

ETHICS AND DISSEMINATION

Research addressing ELGA infants is crucial in order to achieve knowledge of possible causes underlying the development of severe complications and finding preventive strategies. Intervention studies are needed to refute or confirm the suggested effect of human milk-based nutrient fortification. We do not perceive any major health hazard with the study design. Human milk-based nutrient fortifiers are considered to be safe in ELGA infants and safety will be closely monitored.

A written informed consent will be obtained from legal guardians before inclusion. Infants or their families have no specific benefit of study participation besides the possible effects of the human milk-based nutrient fortifier in the active group. Besides the supplementation of the breast milk fortifier and the samples collected in the trial, the participating infants will not be treated differently compared to routine care. The potential benefit for future ELGA infants, however, could be substantial. Finding an intervention that reduces NEC, sepsis and mortality would have a major impact of the well-being in this patient population. It may also elicit beneficial effects on health economics. Only randomised clinical trials will give sufficient evidence for a general recommendation of a new treatment.

Personal information about enrolled participants will be collected, shared and maintained in accordance with the EU General Data Protection Regulation (GDPR).

Crown Princess Victoria Children´s Hospital, County of Östergötland, Linköping, Sweden, is the sponsor and owns all the information obtained in the trial together with the co-ordinating principal investigator. The study is made in collaboration with the company Prolacta Bioscience producing the human milk-based fortifier, which is, for this study, contributed by Prolacta. The study, however, is investigator-initiated. There are no publication restrictions. Study results will be presented at relevant conferences and submitted to peer-reviewed journals. None of the investigators have a financial interest in Prolacta Bioscience. The donating mother signs an informed consent form stating the rights and responsibilities of the donating mother as well as the payment for the provided breast milk. Only excess breast milk, produced by the donating mother, that is beyond the consumptive needs of her nursing child, is accepted.

The potential benefits are considered to outweigh the possible discomfort to the infants and their families. The study is conducted according to ICH/GCP guidelines and was approved by the regional ethical review board at Linköping University (Dnr 2018/193-31, Dnr 2018/384-32).

Acknowledgement

The authors wish to thank all participating hospitals and staff members and the Swedish patient organisation Svenska Prematurförbundet for their invaluable contributions in the planning and realisation of the study.

Authors´ contributions

Co-ordinating principal investigator, TA; Conceptualisation, TA, FA, MD, AE; Formal analysis, TA. MD; Funding acquisition, TA; Methodology, TA, FA, MD, AE; Project administration (steering committee), TA, FA, GBJ, MD, AE, LN; Resources, TA, FA, MD, AE. LN; Supervision TA; Validation, TA, FA, MD, AE. LN; Writing – original draft, GBJ; Writing – review and editing, TA, FA, GBJ, MD, AE, LN.

Funding

For peer review only - http://bmjopen.bmj.com/site/about/guidelines.xhtml

This work is funded by grants from the Swedish Research Council (2020-01111 and 2019-01005), the Research Council for Southeast Sweden, ALF Grants, Prolacta Bioscience, CA, USA.

Competing interests

TA has got a grant for the present study by Prolacta Bioscience, CA, USA, but none of the investigators have any financial interest in Prolacta Bioscience.

For peer review only

REFERENCES

1. Norman, M., et al., *Association Between Year of Birth and 1-Year Survival Among Extremely Preterm Infants in Sweden During 2004-2007 and 2014-2016.* JAMA, 2019. **321**(12): p. 1188-1199.
2. Breastfeeding, A.A.o.P.S.o., *Breastfeeding and the use of human milk.* Pediatrics, 2012. **129**(3): p. e827-41.
3. Underwood, M.A., *Human milk for the premature infant.* Pediatr Clin North Am, 2013. **60**(1): p. 189-207.
4. Nutrition, E.C.o., et al., *Donor human milk for preterm infants: current evidence and research directions.* J Pediatr Gastroenterol Nutr, 2013. **57**(4): p. 535-42.
5. Group, E., *Incidence of and risk factors for neonatal morbidity after active perinatal care: extremely preterm infants study in Sweden (EXPRESS).* Acta Paediatr, 2010. **99**(7): p. 978-92.
6. Lin, P.W. and B.J. Stoll, *Necrotising enterocolitis.* The Lancet, 2006. **368**(9543): p. 1271-1283.
7. Nino, D.F., C.P. Sodhi, and D.J. Hackam, *Necrotizing enterocolitis: new insights into pathogenesis and mechanisms.* Nat Rev Gastroenterol Hepatol, 2016. **13**(10): p. 590-600.
8. Eaton, S., C.M. Rees, and N.J. Hall, *Current Research on the Epidemiology, Pathogenesis, and Management of Necrotizing Enterocolitis.* Neonatology, 2017. **111**(4): p. 423-430.
9. Roze, E., et al., *Functional impairments at school age of children with necrotizing enterocolitis or spontaneous intestinal perforation.* Pediatr Res, 2011. **70**(6): p. 619-25.
10. Humberg, A., et al., *Surgical necrotizing enterocolitis but not spontaneous intestinal perforation is associated with adverse neurological outcome at school age.* Sci Rep, 2020. **10**(1): p. 2373.
11. Ganapathy, V., J.W. Hay, and J.H. Kim, *Costs of necrotizing enterocolitis and cost-effectiveness of exclusively human milk-based products in feeding extremely premature infants.* Breastfeed Med, 2012. **7**(1): p. 29-37.
12. Ganapathy, V., et al., *Long term healthcare costs of infants who survived neonatal necrotizing enterocolitis: a retrospective longitudinal study among infants enrolled in Texas Medicaid.* BMC Pediatr, 2013. **13**: p. 127.
13. Quigley, M., N.D. Embleton, and W. McGuire, *Formula versus donor breast milk for feeding preterm or low birth weight infants.* Cochrane Database Syst Rev, 2019. **7**: p. CD002971.
14. Narayanan, I., et al., *Partial supplementation with expressed breast-milk for prevention of infection in low-birth-weight infants.* Lancet, 1980. **2**(8194): p. 561-3.
15. Narayanan, I., K. Prakash, and V.V. Gujral, *The value of human milk in the prevention of infection in the high-risk low-birth-weight infant.* J Pediatr, 1981. **99**(3): p. 496-8.
16. Narayanan, I., et al., *Randomised controlled trial of effect of raw and holder pasteurised human milk and of formula supplements on incidence of neonatal infection.* Lancet, 1984. **2**(8412): p. 1111-3.
17. Corpeleijn, W.E., et al., *Intake of own mother's milk during the first days of life is associated with decreased morbidity and mortality in very low birth weight infants during the first 60 days of life.* Neonatology, 2012. **102**(4): p. 276-81.

For peer review only

18. Horta, B.L., C. Loret de Mola, and C.G. Victora, *Breastfeeding and intelligence: a systematic review and meta-analysis.* Acta Paediatr, 2015. **104**(467): p. 14-9.
19. Horta, B.L., C. Loret de Mola, and C.G. Victora, *Long-term consequences of breastfeeding on cholesterol, obesity, systolic blood pressure and type 2 diabetes: a systematic review and meta-analysis.* Acta Paediatr, 2015. **104**(467): p. 30-7.
20. Singhal, A., et al., *Breastmilk feeding and lipoprotein profile in adolescents born preterm: follow-up of a prospective randomised study.* Lancet, 2004. **363**(9421): p. 1571-8.
21. Singhal, A., T.J. Cole, and A. Lucas, *Early nutrition in preterm infants and later blood pressure: two cohorts after randomised trials.* Lancet, 2001. **357**(9254): p. 413-9.
22. Singhal, A., et al., *Low nutrient intake and early growth for later insulin resistance in adolescents born preterm.* Lancet, 2003. **361**(9363): p. 1089-97.
23. Fewtrell, M.S., et al., *Early diet and peak bone mass: 20 year follow-up of a randomized trial of early diet in infants born preterm.* Bone, 2009. **45**(1): p. 142-9.
24. Lucas, A., et al., *Breast milk and subsequent intelligence quotient in children born preterm.* Lancet, 1992. **339**(8788): p. 261-4.
25. Anderson, J.W., B.M. Johnstone, and D.T. Remley, *Breast-feeding and cognitive development: a meta-analysis.* Am J Clin Nutr, 1999. **70**(4): p. 525-35.
26. Isaacs, E.B., et al., *Impact of breast milk on intelligence quotient, brain size, and white matter development.* Pediatr Res, 2010. **67**(4): p. 357-62.
27. Ehrenkranz, R.A., et al., *Growth in the neonatal intensive care unit influences neurodevelopmental and growth outcomes of extremely low birth weight infants.* Pediatrics, 2006. **117**(4): p. 1253-61.
28. Lucas, A., J. Boscardin, and S.A. Abrams, *Preterm Infants Fed Cow's Milk-Derived Fortifier Had Adverse Outcomes Despite a Base Diet of Only Mother's Own Milk.* Breastfeed Med, 2020. **15**(5): p. 297-303.
29. Sullivan, S., et al., *An exclusively human milk-based diet is associated with a lower rate of necrotizing enterocolitis than a diet of human milk and bovine milk-based products.* J Pediatr, 2010. **156**(4): p. 562-7.e1.
30. Cristofalo, E.A., et al., *Randomized trial of exclusive human milk versus preterm formula diets in extremely premature infants.* J Pediatr, 2013. **163**(6): p. 1592-1595 e1.
31. O'Connor, D.L., et al., *Nutrient enrichment of human milk with human and bovine milk-based fortifiers for infants born weighing <1250 g: a randomized clinical trial.* Am J Clin Nutr, 2018. **108**(1): p. 108-116.
32. Pocock, S.J. and R. Simon, *Sequential treatment assignment with balancing for prognostic factors in the controlled clinical trial.* Biometrics, 1975. **31**(1): p. 103-15.
33. Bell, M.J., et al., *Neonatal necrotizing enterocolitis. Therapeutic decisions based upon clinical staging.* Ann Surg, 1978. **187**(1): p. 1-7.
34. Gastmeier, P., et al., *Development of a surveillance system for nosocomial infections: the component for neonatal intensive care units in Germany.* J Hosp Infect, 2004. **57**(2): p. 126-31.
35. Hair, A.B., et al., *Beyond Necrotizing Enterocolitis Prevention: Improving Outcomes with an Exclusive Human Milk-Based Diet.* Breastfeeding medicine : the official journal of the Academy of Breastfeeding Medicine, 2016.

For peer review only

36. Sullivan, S., et al., *An exclusively human milk-based diet is associated with a lower rate of necrotizing enterocolitis than a diet of human milk and bovine milk-based products.* The Journal of pediatrics, 2010. **156**(4): p. 562-7 e1.
37. Cristofalo, E.A., et al., *Randomized Trial of Exclusive Human Milk versus Preterm Formula Diets in Extremely Premature Infants.* The Journal of pediatrics, 2013.
38. Gould, A.L., *Interim analyses for monitoring clinical trials that do not materially affect the type I error rate.* Stat Med, 1992. **11**(1): p. 55-66.
39. Pantiadosi, S., *Clinical trials: a methodological perspective, 2nd ed, p 384-386. John Wiley & sons, New Jersey, 2005.* .
40. Haybittle, J.L., *Repeated assessment of results in clinical trials of cancer treatment.* Br J Radiol, 1971. **44**(526): p. 793-7.
41. Rochow, N., et al., *Target fortification of breast milk: how often should milk analysis be done?* Nutrients, 2015. **7**(4): p. 2297-310.
42. Jobe, A.H. and E. Bancalari, *Bronchopulmonary dysplasia.* Am J Respir Crit Care Med, 2001. **163**(7): p. 1723-9.
43. International Committee for the Classification of Retinopathy of, P., *The International Classification of Retinopathy of Prematurity revisited.* Arch Ophthalmol, 2005. **123**(7): p. 991-9.
44. Papile, L.A., et al., *Incidence and evolution of subependymal and intraventricular hemorrhage: a study of infants with birth weights less than 1,500 gm.* J Pediatr, 1978. **92**(4): p. 529-34.
45. de Vries, L.S., P. Eken, and L.M. Dubowitz, *The spectrum of leukomalacia using cranial ultrasound.* Behav Brain Res, 1992. **49**(1): p. 1-6.
46. Bayley, N., *Bayley Scales of Infant and Toddler Development*. 3rd ed. 2006, San Antonio, TX: Harcourt Assessment Inc.

For peer review only

For peer review only - http://bmjopen.bmj.com/site/about/guidelines.xhtml

TABLES

Table 1 Inclusion and exclusion criteria

Inclusion criteria	
	· Gestational age at birth 22+0-27+6; based on prenatal ultrasonography<br>· Enteral feeds < 100 mL/kg/day at the day of randomisation<br>· Written informed consent from the legal guardians of the infant<br>· The home clinic of the infant has the logistics of maintaining the intervention until week 34+0
Exclusion criteria	
	· Lethal or complicated malformation known at the time of inclusion<br>· Chromosomal anomalies known at the time of inclusion<br>· No realistic hope for survival at the time of inclusion<br>· Gastrointestinal malformation known at the time of inclusion<br>· Abdominal surgery before the time of inclusion<br>· Participation in another intervention trial aiming at having an effect on growth, nutrition, feeding intolerance or severe complications such as NEC and sepsis<br>· Infants having nutrient fortifier or formula prior to randomisation

NEC, necrotising enterocolitis.

For peer review only

Table 2 Clinical outcomes

	Description	Time Frame
Primary clinical outcome		
Composite of NEC, culture-proven sepsis and mortality	Requires any of these three diagnoses to fulfil the criterion	A
Secondary clinical outcomes		
Feeding intolerance and growth		
Time to reach full enteral feeds	The day of life the infant has received at least 150 mL/kg enteral feeds	A
Feeding interruption	Number of days feedings held for ≥12 hours or feeds reduced by >50% (mL/kg/d) not due to a clinical procedure or transitioning to the breast	A
Parenteral nutrition	Number of days of parental amino acid and/or lipid infusion; only days when the enteral feed <150 mL/kg/day should be included	A
Gastric aspirates	≥100% of pre-feed volume (2 hours volume if continuous); lower limit of 2 mL/kg	A
Stool frequency	Number of stools per day	A
Time to regain birth weight	First day of three success days when the infant weight is greater than birth weight	A
Weight, height and head circumference	Standard deviation score will be used for the calculations	B
Clinical variables for morbidity		
Infancy		
Mortality		A
Necrotising enterocolitis (NEC)	Bell´s stage II-III [33]; radiological assessment will be made blinded by an independent radiologist; the final decision will be confirmed by a blinded consensus panel review consisting of the investigators	A
Culture-proven sepsis	Positive blood- and/or urine- and/or CSF culture, clinical deterioration and laboratory inflammatory response [34]; further classified into early (<72 hours postpartum) or late (>72 hours postpartum) onset.	A
Composite of NEC and culture-proven sepsis	Requires any of these two diagnoses to fulfil the criterion	A
Mortality and morbidity index (MMI)	Composite measure requiring any of the following: death, NEC stage II-III, culture-proven sepsis, moderate to severe BPD or ROP stage III-V	
Spontaneous intestinal perforation (SIP)	Intestinal perforation without signs of intramural and/or portal gas and no signs of inflammation at surgery	A
Abdominal surgery		A
Suspected sepsis, not culture-proven	Clinical deterioration and laboratory inflammatory response but negative blood culture	A
Pneumonia	Pathological X-ray confirmed by a radiologist, need of increased respiratory support/oxygen and laboratory inflammatory response	A
Bronchopulmonary dysplasia (BPD)	Need of extra oxygen, high flow nasal cannula, CPAP or ventilator at w36+0 [42]	C
Retinopathy of prematurity (ROP)	Diagnosed by an independent ophthalmologist according to international classification; classified into stage I-V [43]; the diagnosis is set after w42+0	D
Intraventricular haemorrhage (IVH)	Assessed with ultrasound; classified into grade I-IV [44]	A
Periventricular leukomalacia (PVL)	Assessed with ultrasound and MRI; criteria according to de Vries [45]	A
Number of days with intensive care	Need of respirator or CPAP	A
Length of stay at the hospital	GW and day at discharge	A
Length of need of feeding tube	GW and day when the infant does not need it anymore	A
2-year follow-up		
Neurocognitive development	Bayleys III tested by psychologist [46]	E
Cerebral palsy	Tested by an experienced paediatrician and/or physiotherapist	E
Epilepsy		E
Strabismus and/or impaired vision		E
Impaired hearing		E
Respiratory support	Need of extra oxygen and/or ventilatory support	F
Wheeze and/or asthma		G
Severe infections		H
Mortality	Including cause of death	H
Need of feeding tube		H

For peer review only

Extra nutritional support		H
Level of education of the parents		E
Family status		E
Day-care		E
5.5-year follow up		
Neurocognitive development	Wechsler Preschool and Primary Scale of Intelligence IV (WPPSI-IV) tested by a psychologist and Movement ABC-2 tested by a physiotherapist	I
Cerebral palsy	Tested by an experienced paediatrician and/or physiotherapist	I
Epilepsy		I
Strabismus and/or impaired vision		I
Impaired hearing		I
Wheeze and/or asthma		I
Mortality	Including cause of death	J

Timeframe **A**, from birth until discharge (no longer than w44+0); **B**, at 7, 14, 21 and 28 days, end of intervention (≤ w34+0), w36+0, discharge (no longer than w44+0), and 2 years of age (corrected) and 5.5 years of age (uncorrected); **C**, at w36+0; **D**, from birth until w42+0; **E**, at 2 years of age (corrected); **F**, from w44+0 until 2 years of age; **G**, from birth until 2 years of age; **H**, from discharge or w44+0 (whether comes first) until 2 years of age; **I**, at 5.5 years of age (uncorrected); **J**, from 2 years until 5 years of age. Week (w) refers to postmenstrual week.

For peer review only

For peer review only - http://bmjopen.bmj.com/site/about/guidelines.xhtml

Table 3 Associated predictor variables (covariates)

1	Gender
2	Caesarean section
3	Multiple pregnancies
4	Birth weight and height
5	Small for gestational age (SGA), birth weight <2 SD
6	Maternal smoking during pregnancy
7	Preeclampsia, diagnosis by the responsible obstetrician
8	Chorioamnionitis, clinical diagnosis by the responsible obstetrician
9	Preterm premature rupture of membranes (PPROM), rupture > 1 hour before contractions started
10	Antenatal antibiotics, pertaining the period of the mother´s actual attendance at the hospital
11	Antenatal corticosteroids; the mother should have received at least 12 mg betamethasone. The corticosteroid prophylaxis is considered completed if the mother has received 2 doses at least 24 hours before delivery
12	Born at level 1-2 NICUs
13	NICU inclusion site
14	Apgar score
15	Surfactant-administration
16	Intubation
17	Infant respiratory distress syndrome (IRDS), verified by X-ray
18	Mechanical ventilation, duration
19	Patent ductus arteriosus (PDA), requiring medical or surgical treatment
20	Antibiotics, drug, treatment period and number of days
21	Probiotics, name, treatment period and number of days
22	Opioids, drug, treatment period and number of days
23	Gastric acid inhibitors, drug, treatment period and number of days
24	Day of life when the supplementation of the study product was started
25	The amount of enteral feeds per day that the infants received when the supplementation of the study product was started
26	Number of days the infant has not received the study product
27	Intravenous lines, number of days
28	Insulin treatment, number of days
29	Postnatal corticosteroids, number of days
30	Inotropic drugs, number of days
31	Feeding regime, continuous or bolus
32	Amount of nutrient protein fortifier per day
33	Amount of fat supplement per day
34	Total amount of protein, fat, carbohydrate, energy and micronutrient per day
35	The relative amount of donor breast milk per day
36	Amount of extra enteral lipid
37	Breastfeeding, exclusive or partial

NICU, neonatal intensive care unit.

For peer review only

Table 4 Expected severe adverse events (SAE)

Severe infection such as pneumonia, sepsis or meningitis
CMV infection, upper respiratory viral infection
NEC, SIP and/or need of abdominal surgery
Respiratory distress syndrome (RDS) and BPD
Intracranial bleeding, PVL or hydrocephalus
Lung bleeding
Pneumothorax, pleural effusion
Pulmonary hypertension
Persistent ductus arteriosus (PDA)
Retinopathy of prematurity (ROP)
Death

CMV, cytomegalovirus; NEC, necrotising enterocolitis; SIP, spontaneous intestinal perforation; BPD, bronchopulmonary dysplasia; PVL, periventricular leukomalacia

For peer review only

Figure 1 Summary of the N-Forte study actions

	Neonatal period									Follow-up	
**Time point**	**Birth**	**<100 mL/kg/d**	**7±2 d**	**14±2 d**	**21±2 d**	**28±2 d**	**w34+0**	**w36+0**	**Discharge***	**2 years**	**5.5 years**
Enrolment											
Information given		X									
Eligibility screen		X									
Informed consent		X									
Allocation		X									
Perinatal data	X										
Intervention		◆	—	—	—	—	◆				
Feeding intolerance		◆	—	—	—	—	◆				
NEC, sepsis mortality		◆	—	—	—	—	—	—	◆		
Clinical data noted in the CRF on a daily basis	◆	—	—	—	—	—	—	—	◆		
Neurodevelopmental impairment										X	X
Antibiotics and concomitant medication		X	X	X	X	X	X	X	X		
Weight, length and head circumference**	X		X	X	X	X	X	X	X	X	X
Blood sample			X	X		X	w33+0-6				
Urine sample			X	X	X	X	w33+0-6				
Stool sample			X	X	X	X	w33+0-6				
Breast milk sample			X	X	X	X	w33+0-6				
Adverse events		X	X	X	X	X	X	X	X		

Week (w) refers to postmenstrual week. *Discharge or at latest postmenstrual week 44+0. **Only indicated when measurements must be made in the trial (weight is often measured every day).

For peer review only

Supplementary table 1 Nutritional content of the study product

Nutrient	Humavant+6®	Humavant CR®	Unit
Calories	143.82	262.22	kcal/100 mL
Fat	9.38	25.59	g/100 mL
Protein	5.92	0.82	g/100 mL
Carbohydrates	8.91	6.92	g/100 mL
Saturated fat	3.93	10.15	g/100 mL
Sugar	6.12	4.53	g/100 mL
Vitamins			
Vit A	46.21	114.65	µg/100 mL
Vit D	0.21	0.09	µg/100 mL
Vit K	<5.10	<4.85	µg/100 mL
Vit C	<1.02	<0.97	mg/100 mL
Vit B1, thiamine	5.92	<4.85	µg/100 mL
Vit B2, riboflavin	19.18	11.14	µg/100 mL
Vit B6, pyridoxine	<5.10	<4.85	µg/100 mL
Niacin	0.08	0.06	mg/100 mL
Folic acid	13.06	<2.43	µg/100 mL
Vit B12, cobalamin	<0.14	<0.14	µg/100 mL
Pantothenic acid	0.17	0.15	mg/100 mL
Biotin	<2.55	<3.17	µg/100 mL
Vit E, α-tocopherol	0.37	1.54	mg/100 mL
Minerals			
Salts	326.10	17.27	mg/100 mL
Sodium	130.69	6.77	mg/100 mL
Chloride	101.79	N/A	mg/100 mL
Potassium	187.17	64.83	mg/100 mL
Calcium	339.86	19.82	mg/100 mL
Phosphorus	189.20	2.83	mg/100 mL
Magnesium	21.48	0.02	mg/100 mL
Iron	0.13	0.33	mg/100 mL
Zinc	2.28	0.03	mg/100 mL
Copper	237.50	<0.03	µg/100 mL
Iodine	23.36	19.89	µg/100 mL
Selenium	9.65	2.07	µg/100 mL
Manganese	5.11	<4.03	µg/100 mL
Chromium	1.57	<0.41	µg/100 mL
Molybdenum	2.55	<1.94	µg/100 mL
Fluoride	<0.02	<0.02	mg/100 mL

For peer review only

STANDARD PROTOCOL ITEMS: RECOMMENDATIONS FOR INTERVENTIONAL TRIALS

SPIRIT 2013 Checklist: Recommended items to address in a clinical trial protocol and related documents*

Section/item	Item No	Description<br>*Marked in red are the relevant page references to the main document and supplementary material*
**Administrative information**		
Title	1	Descriptive title identifying the study design, population, interventions, and, if applicable, trial acronym<br>*Page 1*
Trial registration	2a	Trial identifier and registry name. If not yet registered, name of intended registry<br>*Page 2*
	2b	All items from the World Health Organization Trial Registration Data Set
Protocol version	3	Date and version identifier<br>*Page 5*
Funding	4	Sources and types of financial, material, and other support<br>*Page 10*
Roles and responsibilities	5a	Names, affiliations, and roles of protocol contributors<br>*Page 1 (names, affiliations); Page 10 (roles/contributions)*
	5b	Name and contact information for the trial sponsor<br>*Page 10*
	5c	Role of study sponsor and funders, if any, in study design; collection, management, analysis, and interpretation of data; writing of the report; and the decision to submit the report for publication, including whether they will have ultimate authority over any of these activities<br>*Page 10*
	5d	Composition, roles, and responsibilities of the coordinating centre, steering committee, endpoint adjudication committee, data management team, and other individuals or groups overseeing the trial, if applicable (see Item 21a for data monitoring committee)<br>*Page 10*
**Introduction**		

For peer review only

Background and rationale	6a	Description of research question and justification for undertaking the trial, including summary of relevant studies (published and unpublished) examining benefits and harms for each intervention<br>*Pages 3 to 4*
	6b	Explanation for choice of comparators<br>*Page 9*
Objectives	7	Specific objectives or hypotheses<br>*Page 4*
Trial design	8	Description of trial design including type of trial (eg, parallel group, crossover, factorial, single group), allocation ratio, and framework (eg, superiority, equivalence, noninferiority, exploratory)<br>*Page 5*

Methods: Participants, interventions, and outcomes

Study setting	9	Description of study settings (eg, community clinic, academic hospital) and list of countries where data will be collected. Reference to where list of study sites can be obtained<br>*Page 5*
Eligibility criteria	10	Inclusion and exclusion criteria for participants. If applicable, eligibility criteria for study centres and individuals who will perform the interventions (eg, surgeons, psychotherapists)<br>*Pages 5 and 16 (Table 1)*
Interventions	11a	Interventions for each group with sufficient detail to allow replication, including how and when they will be administered<br>*Pages 5 to 6 and Supplementary table 1*
	11b	Criteria for discontinuing or modifying allocated interventions for a given trial participant (eg, drug dose change in response to harms, participant request, or improving/worsening disease)<br>*Page 6*
	11c	Strategies to improve adherence to intervention protocols, and any procedures for monitoring adherence (eg, drug tablet return, laboratory tests)<br>*N/A*
	11d	Relevant concomitant care and interventions that are permitted or prohibited during the trial<br>*Page 6 ("The infants in neither group must not be fed with formula during the intervention period")*

For peer review only - http://bmjopen.bmj.com/site/about/guidelines.xhtml

Outcomes	12	Primary, secondary, and other outcomes, including the specific measurement variable (eg, systolic blood pressure), analysis metric (eg, change from baseline, final value, time to event), method of aggregation (eg, median, proportion), and time point for each outcome. Explanation of the clinical relevance of chosen efficacy and harm outcomes is strongly recommended<br>*Pages 6 to 7, 15 (Figure 1), 17 to 18 (Table 2), and 19 (Table 3)*
Participant timeline	13	Time schedule of enrolment, interventions (including any run-ins and washouts), assessments, and visits for participants. A schematic diagram is highly recommended (see Figure)<br>*Pages 5 to 6 and 15 (Figure 1)*
Sample size	14	Estimated number of participants needed to achieve study objectives and how it was determined, including clinical and statistical assumptions supporting any sample size calculations<br>*Pages 7 to 8*
Recruitment	15	Strategies for achieving adequate participant enrolment to reach target sample size<br>*Page 5 (Setting and participants)*

Methods: Assignment of interventions (for controlled trials)

Allocation:

Sequence generation	16a	Method of generating the allocation sequence (eg, computer-generated random numbers), and list of any factors for stratification. To reduce predictability of a random sequence, details of any planned restriction (eg, blocking) should be provided in a separate document that is unavailable to those who enrol participants or assign interventions<br>*Page 5*
Allocation concealment mechanism	16b	Mechanism of implementing the allocation sequence (eg, central telephone; sequentially numbered, opaque, sealed envelopes), describing any steps to conceal the sequence until interventions are assigned<br>*Page 5*
Implementation	16c	Who will generate the allocation sequence, who will enrol participants, and who will assign participants to interventions<br>*Page 5*
Blinding (masking)	17a	Who will be blinded after assignment to interventions (eg, trial participants, care providers, outcome assessors, data analysts), and how<br>*Page 9 ("The radiological assessment of NEC will be made blinded by an independent radiologist. The secondary outcome ROP will be diagnosed by an independent ophthalmologist.")*

	17b	If blinded, circumstances under which unblinding is permissible, and procedure for revealing a participant's allocated intervention during the trial<br>*N/A*

Methods: Data collection, management, and analysis

Data collection methods	18a	Plans for assessment and collection of outcome, baseline, and other trial data, including any related processes to promote data quality (eg, duplicate measurements, training of assessors) and a description of study instruments (eg, questionnaires, laboratory tests) along with their reliability and validity, if known. Reference to where data collection forms can be found, if not in the protocol<br>*Pages 6 to 7*
	18b	Plans to promote participant retention and complete follow-up, including list of any outcome data to be collected for participants who discontinue or deviate from intervention protocols<br>*Page 6*
Data management	19	Plans for data entry, coding, security, and storage, including any related processes to promote data quality (eg, double data entry; range checks for data values). Reference to where details of data management procedures can be found, if not in the protocol<br>*Page 7*
Statistical methods	20a	Statistical methods for analysing primary and secondary outcomes. Reference to where other details of the statistical analysis plan can be found, if not in the protocol<br>*Page 8*
	20b	Methods for any additional analyses (eg, subgroup and adjusted analyses)<br>*Page 8*
	20c	Definition of analysis population relating to protocol non-adherence (eg, as randomised analysis), and any statistical methods to handle missing data (eg, multiple imputation)<br>*N/A*

Methods: Monitoring

Data monitoring	21a	Composition of data monitoring committee (DMC); summary of its role and reporting structure; statement of whether it is independent from the sponsor and competing interests; and reference to where further details about its charter can be found, if not in the protocol. Alternatively, an explanation of why a DMC is not needed<br>*Page 7*

	21b	Description of any interim analyses and stopping guidelines, including who will have access to these interim results and make the final decision to terminate the trial<br>*Pages 7 to 8*
Harms	22	Plans for collecting, assessing, reporting, and managing solicited and spontaneously reported adverse events and other unintended effects of trial interventions or trial conduct<br>*Pages 7 and 20 (Table 4)*
Auditing	23	Frequency and procedures for auditing trial conduct, if any, and whether the process will be independent from investigators and the sponsor<br>*Page 7*
**Ethics and dissemination**		
Research ethics approval	24	Plans for seeking research ethics committee/institutional review board (REC/IRB) approval<br>*N/A (Already approved by the regional ethical review board in Linköping Sweden (Dnr 2018/193-31, Dnr 2018/384-32)*
Protocol amendments	25	Plans for communicating important protocol modifications (eg, changes to eligibility criteria, outcomes, analyses) to relevant parties (eg, investigators, REC/IRBs, trial participants, trial registries, journals, regulators)<br>*N/A*
Consent or assent	26a	Who will obtain informed consent or assent from potential trial participants or authorised surrogates, and how (see Item 32)<br>*Page 5*
	26b	Additional consent provisions for collection and use of participant data and biological specimens in ancillary studies, if applicable<br>*Supplementary material (Parent Information and consent)*
Confidentiality	27	How personal information about potential and enrolled participants will be collected, shared, and maintained in order to protect confidentiality before, during, and after the trial<br>*Supplementary material (Parent Information and consent)*
Declaration of interests	28	Financial and other competing interests for principal investigators for the overall trial and each study site<br>*Page 11*
Access to data	29	Statement of who will have access to the final trial dataset, and disclosure of contractual agreements that limit such access for investigators<br>*Page 10*

For peer review only

Ancillary and post-trial care	30	Provisions, if any, for ancillary and post-trial care, and for compensation to those who suffer harm from trial participation<br>*N/A*
Dissemination policy	31a	Plans for investigators and sponsor to communicate trial results to participants, healthcare professionals, the public, and other relevant groups (eg, via publication, reporting in results databases, or other data sharing arrangements), including any publication restrictions<br>*Pages 2, 10, and supplementary material (Parent Information and consent)*
	31b	Authorship eligibility guidelines and any intended use of professional writers<br>*Page 10*
	31c	Plans, if any, for granting public access to the full protocol, participant-level dataset, and statistical code<br>*N/A*
**Appendices**		
Informed consent materials	32	Model consent form and other related documentation given to participants and authorised surrogates<br>*Supplementary material (Parent Information and consent)*
Biological specimens	33	Plans for collection, laboratory evaluation, and storage of biological specimens for genetic or molecular analysis in the current trial and for future use in ancillary studies, if applicable<br>*Page 7 and supplementary material (Parent Information and consent)*

*It is strongly recommended that this checklist be read in conjunction with the SPIRIT 2013 Explanation & Elaboration for important clarification on the items. Amendments to the protocol should be tracked and dated. The SPIRIT checklist is copyrighted by the SPIRIT Group under the Creative Commons "Attribution-NonCommercial-NoDerivs 3.0 Unported" license.

For peer review only - http://bmjopen.bmj.com/site/about/guidelines.xhtml
